# Antimicrobial Benzyltetrahydroisoquinoline-Derived Alkaloids from the Leaves of *Doryphora aromatica*

**DOI:** 10.1021/acs.jnatprod.0c01093

**Published:** 2021-03-05

**Authors:** Miaomiao Liu, Jianying Han, Yunjiang Feng, Gordon Guymer, Paul I. Forster, Ronald J. Quinn

**Affiliations:** † Griffith Institute for Drug Discovery, Griffith University, Brisbane, QLD 4111, Australia; ¶ Queensland Herbarium, Department of Environment and Science, Brisbane Botanic Gardens, Brisbane, QLD 4066, Australia

## Abstract

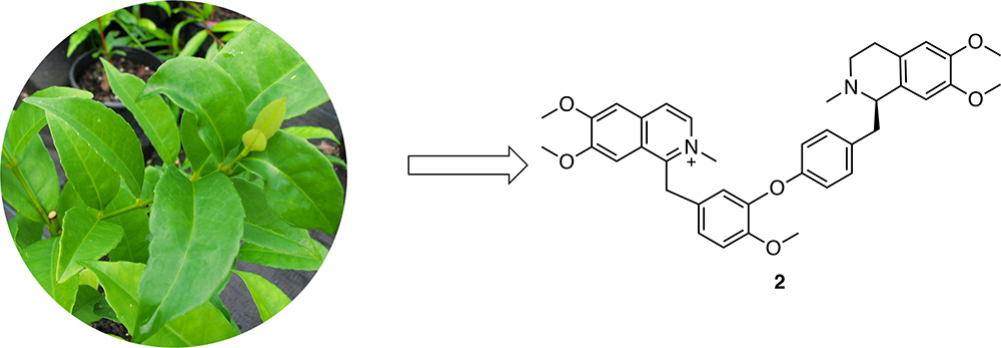

Four new alkaloids, (*R*)-nomimantharine trifluoroacetate (**2**), 12-demethylphaeantharine trifluoroacetate (**3**), nominanthranal trifluoroacetate (**4**), and the enolic form of 1-hydroxy-6,7-dimethoxy-2-methylisoquinoline trifluoroacetate (**5**), together with the known dimeric alkaloid phaeantharine trifluoroacetate (**1**), have been isolated from the extract of the leaves of the rainforest tree *Doryphora aromatica* (Monimiaceae). The structures of these compounds were elucidated by HRMS and 1D and 2D NMR data. (*R*)-Nomimantharine trifluoroacetate (**2**) contains an ether linkage connecting a benzylisoquinoline unit with a tetrahydroisoquinoline, a novel class of dimeric alkaloid. The absolute configuration of (*R*)-nomimantharine trifluoroacetate (**2**) was established via electronic circular dichroism data. The compounds isolated were subjected to in vitro antimicrobial assays against a panel of pathogenic microorganisms, including *Mycobacterium smegmatis*, *M. tuberculosis*, *Escherichia coli*, *Staphylococcus aureus* (SA), and five clinical isolates of oxacillin/methicillin-resistant *S. aureus* (MRSA). Phaeantharine trifluoroacetate (**1**) and (*R*)-nomimantharine trifluoroacetate (**2**) showed moderate inhibitory activities against *Mycobacteria* and MRSA strains.

Since the discovery of penicillin, antibiotics have saved millions of lives every year. However, the advent of drug resistance has rendered many of them ineffective against organisms such as methicillin-resistant *Staphylococcus aureus* (MRSA) and multidrug- and extensively drug-resistant *Mycobacterium tuberculosis*.^1,2^ New drugs that retain potency against multidrug/extensively drug-resistant strains with the additional benefit of a shortened treatment duration and ease of administration are urgently needed. Natural products (NPs) are superior starting points for the major antimicrobials used in the clinic.^3,4^ Antimicrobial NPs can be obtained from microorganisms and plants.^5−9^

The traditional process of discovering new bioactive natural products via bioassay-guided isolation is generally long and laborious, and known natural products are frequently rediscovered. This work reports a combination of HPLC fractionation and an NMR fingerprint technique to discover compounds with new structures. We have previously reported a strategy to generate a natural product fraction library with lead- and drug-like constituents by selecting favorable physicochemical properties such as logP < 5.^10,11^ The whole library (202 983 fractions) was evaluated by a phenotypic high-throughput screening (HTS) assay against *M. tuberculosis* H37Rv and resulted in a series of active fractions. As part of a strategy to build an NMR database of fractions, 748 biota, including 291 marine organisms and 457 plant samples, were re-extracted and refractionated with collection of five fractions per extract to give 3740 fractions according to the reported protocol.^10^ Each fraction in the library was evaluated by comparison of NMR spectra to achieve NMR fingerprinting to aid the identification of new constituents (Figure 1). The NMR spectrum of a fraction is a fingerprint of its entire chemical composition and, therefore, never lies about the composition of fractions.^12^ The aim of NMR fingerprinting-guided NP discovery is to detect as many compounds as possible, which means maintaining high detection sensitivity. The advent of high-field instruments together with cryoprobes and small volume tubes (3 or 1.7 mm NMR tubes) has addressed the previous limitation of low sensitivity so that NMR spectra of fractions can be directly analyzed to identify constituents. Since most secondary metabolites contain hydrogen, the ^1^H NMR spectrum of samples containing a large number of metabolites would result in extensive overlap in signals in the NMR fingerprint. The NMR fingerprint of fractions gave spectra with sufficient resolution to allow compound isolation to be monitored with the objective to isolate all constituents in the fraction by ensuring all signals in the fraction were present within one or more of the isolated compounds. With the guidance of NMR fingerprinting, we proceeded to isolate active compounds in one of the prioritized active fractions (Figure 1).

**Figure 1:**
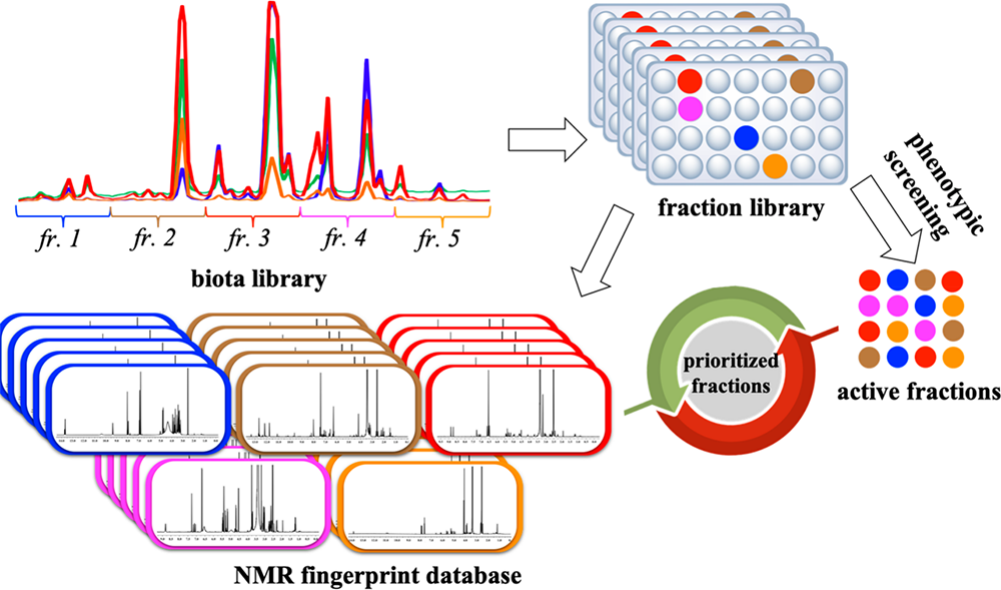
Overview of natural product drug discovery using both phenotypic screening and NMR fingerprinting.

In this study, we report compound isolation and identification from an active fraction prioritized from the phenotypic HTS screening in combination with NMR fingerprinting. The fraction was derived from a sample of the rainforest tree *Doryphora aromatica* (F.M.Bailey) L.S.Sm. (family Monimiaceae), which belongs to the order Laurales sensu Dahlgren and comprises about 28 genera and 195–200 species.^13^ Monimiaceae is a family rich in benzyltetrahydroisoquinoline-derived proaporphine, aporphine, and oxoaporphine alkaloids (Figure 2A).^14,15^ Less common is the presence of morphinanedienone-type compounds, such as flavinantine and saludimerines (Figure 2B).^16,17^ In our current study, five benzyltetrahydroisoquinoline-derived alkaloids, including four new members, were isolated from *Doryphora aromatica*. While bisbenzylisoquinolines (**1**) and bistetrahydroisoquinolines are known, (*R*)-nomimantharine trifluoroacetate (**2**) contains an ether linkage connecting a benzylisoquinoline unit and a tetrahydroisoquinoline unit, a novel class of dimeric alkaloid. Compounds were assayed against a panel of pathogenic microorganisms, and phaeantharine trifluoroacetate (**1**) and (*R*)-nomimantharine trifluoroacetate (**2**) exhibited activities against *Mycobacteria* and MRSA strains.

**Figure 2:**
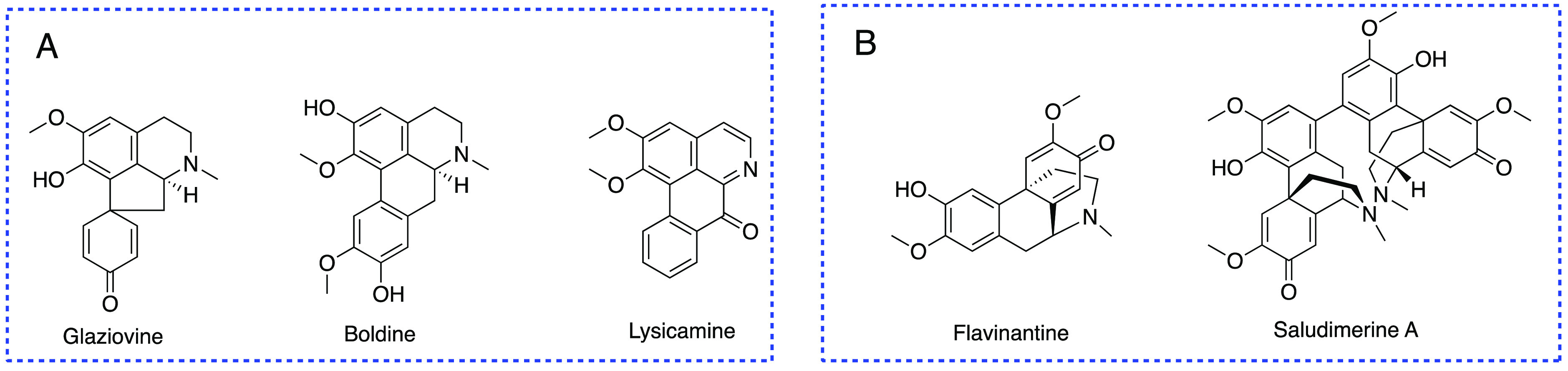
(A) Structures of proaporphine, aporphine, and oxoaporphine alkaloids. (B) Structures of flavinantine and saludimerines.

## Results and Discussion

**Figure 3:**
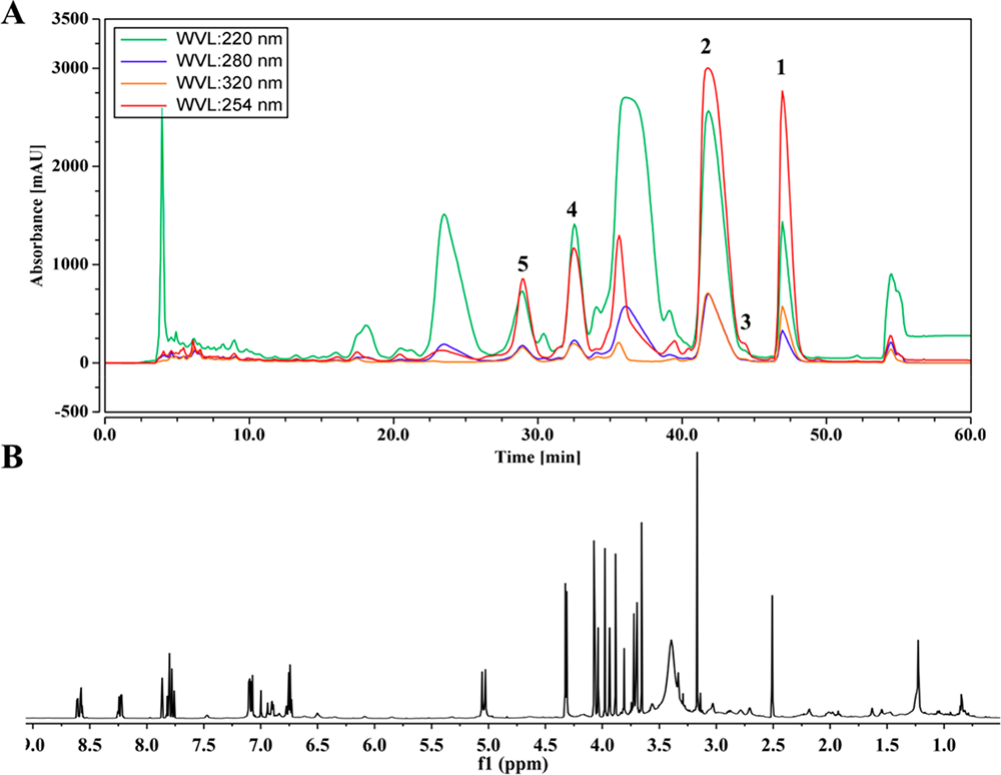
(A) HPLC profile of the alkaloid fraction of *D. aromatica*. (B) NMR fingerprint of the alkaloid fraction of *D. aromatica*.

The leaves of *D. aromatica* were extracted using a 95% EtOH solution followed by Sephadex LH-20 fractionation. Analysis of the NMR fingerprints of all fractions identified an enriched alkaloid fraction. The latter was fractionated by semipreparative HPLC, resulting in the isolation of major compounds **1** and **2** as well as the minor secondary metabolites **3**–**5** (Figure 3).

**Figure 4:**
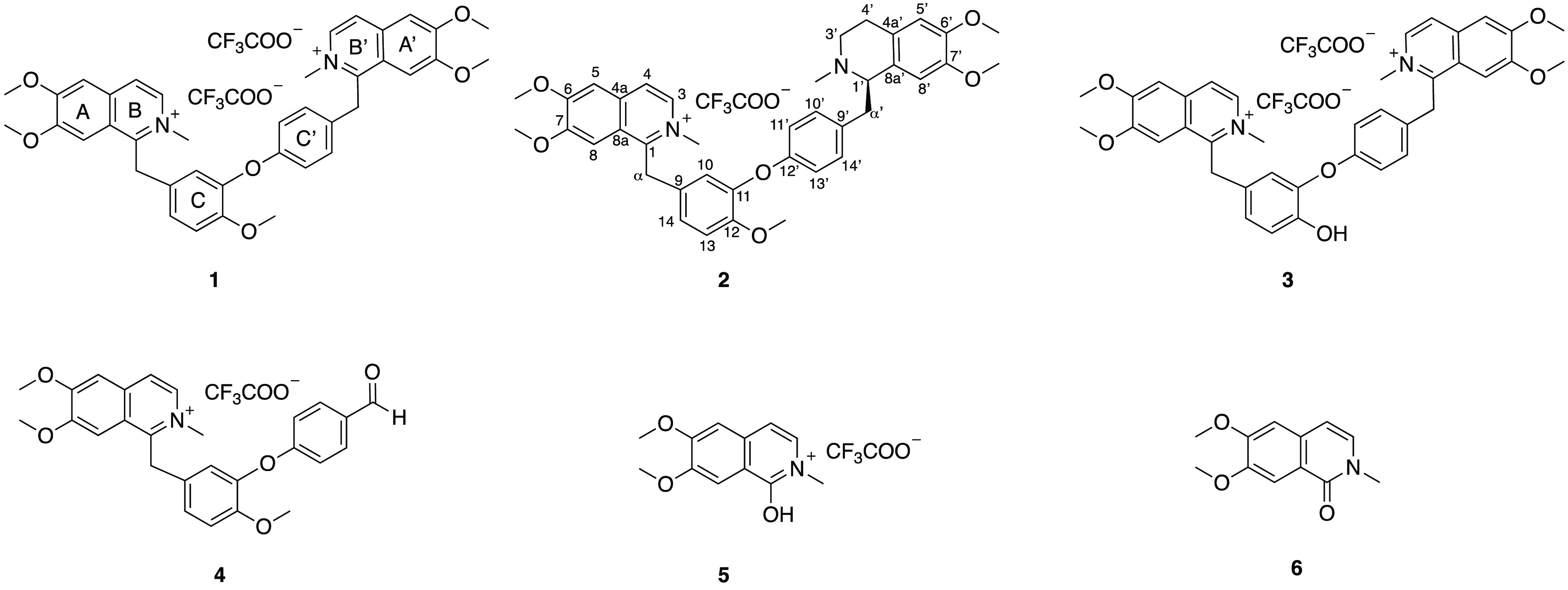
Structures of compounds **1**–**6**.

NMR data (Tables 1 and 2) and 2D NMR experiments suggested that compounds **1**–**5** are closely related, all possessing a 6,7-dimethoxy-2-methylisoquinolinium moiety. ^1^H and ^13^C NMR spectra of compound **1** showed a number of paired signals, indicating a significant level of symmetry. The structure of compound **1** (Figure 4) was unambiguously identified as the TFA salt of the known compound phaeantharine, an alkaloid previously isolated from *Phaeanthus ebracteolatus*, by comparison of the observed and reported data.^18^

**Table 1:** ^**1**^H NMR Data of Compounds **1**–**5** in DMSO-*d*_6_ at 800 MHz

	**1**	**2**	**3**	**4**	**5**
position	δ_H_, mult. (*J* in Hz)	δ_H_, mult. (*J* in Hz)	δ_H_, mult. (*J* in Hz)	δ_H_, mult. (*J* in Hz)	δ_H_, mult. (*J* in Hz)
3	8.51, d (7.0)	8.53, d (7.2)	8.50, d (6.8)	8.53, d (7.2)	7.35, d (7.2)
4	8.20, d (6.8)	8.21, d (7.2)	8.19, d (6.7)	8.21, d (6.7)	6.52, d (7.2)
5	7.73, s	7.73, s	7.73, s	7.74, s	7.15, s
8	7.78, s	7.82, s	7.79, s	7.83, s	7.58, s
α	4.98, s	5.04, d (17.3)	4.93, s	5.04, s	
		4.99, d (17.3)			
10	6.98, d (2.6)	6.95, d (2.4)	6.91, d (1.8)	7.13, d (2.1)	
13	7.07, d (8.4)	7.11, d (8.9)	6.88, d (8.4)	7.16, d (8.8)	
14	6.89, dd (2.1, 8.4)	6.92, dd (2.4, 8.9)	6.74, dd (2.3, 6.8)	7.00, dd (2.1, 8.6)	
1′		4.53 m			
3′	8.53, d (7.2)	3.73, m	8.54, d (6.9)		
		3.39, m			
4′	8.22, d (6.8)	2.99, m	8.22, d (6.7)		
5′	7.76, s	6.85, s	7.76, s		
8′	7.84, s	5.71, s	7.84, s		
α′	5.01, s	3.37, m	5.01, s	9.89, s	
		2.96, m			
10′/14′	7.07, dd (5.2, 8.4)	7.06, d (8.7)	7.08, dd (8.8)	7.86, d (8.7)	
11′/13′	6.74, dd (2.1, 8.4)	6.77, d (8.7)	6.75, dd (1.7, 8.7)	6.95, d (8.7)	
1-OH					6.54 brs
CH_3_N-2	4.27, s	4.30, s	4.28, s	4.32, s	4.32, s
CH_3_O-6	4.06, s	4.04, s	4.06, s	4.07, s	4.07, s
CH_3_O-7	3.87, s	3.93, s	3.88, s	3.94, s	3.94, s
12-OH			9.73, s		
CH_3_O-12	3.64, s	3.69, s		3.69, s	
CH_3_N-2′	4.28, s	2.84, s	4.29, s		
CH_3_O-6′	4.07, s	3.74, s	4.07, s		
CH_3_O-7′	3.96, s	3.29, s	3.96, s		

**Table 2:** ^13^C NMR Data of Compounds **1**–**5** in DMSO-*d*_6_ at 200 MHz

	**1**	**2**	**3**	**4**	**5**
position	δ_C_, type	δ_C_, type	δ_C_, type	δ_C_, type	δ_C_, type
1	155.2, C	155.7, C	156.0, C	155.6, C	161.2, C
3	136.2, CH	136.8, CH	136.4, CH	136.5, CH	132.4, CH
4	122.1, CH	122.7, CH	122.3, CH	122.4, CH	104.9, CH
4a	135.6, C	136.2, C	136.2, C	136.1, C	132.9, C
5	106.4, CH	106.9, CH	106.6, CH	106.8, CH	107.0, CH
6	156.7, C	157.2, C	157.6, C	157.3, C	153.3, C
7	152.6, C	153.2, C	153.2, C	153.1, C	149.2, C
8	106.0, CH	106.4, CH	106.6, CH	106.4, CH	107.2, CH
8a	123.7, C	124.2, C	124.1, C	124.3, C	119.5, C
α	33.0, CH_2_	33.5, CH_2_	33.1, CH_2_	33.5, CH_2_	
9	127.8, C	128.2, C	126.7, C	128.5, C	
10	122.0, CH	122.3, CH	122.8, CH	123.0, CH	
11	143.3, C	144.0, C	142.6, C	142.7, C	
12	150.4, C	151.0, C	149.0, C	150.9, C	
13	114.0, CH	114.5, CH	118.1, CH	114.6, CH	
14	125.4, CH	125.8, CH	125.7, CH	126.7, CH	
1′	155.4, C	64.3, CH	156.0, C		
3′	136.3, CH	44.7, CH_2_	136.4, CH		
4′	122.2, CH	21.3, CH_2_	122.3, CH		
4a′	135.7, C	122.3, C	136.2, C		
5′	106.4, CH	112.1, CH	106.6, CH		
6′	156.8, C	148.9, C	157.6, C		
7′	152.7, C	146.9, C	153.2, C		
8′	105.9, CH	111.6, CH	106.3, CH		
8a′	123.8, C	121.3, C	124.1, C		
α′	33.2, CH_2_	40.2, CH_2_	33.8, CH_2_	191.6, CH	
9′	128.6, C	130.1, C	129.2, C	131.3, C	
10′/14′	129.6, CH	131.7, CH	129.8, CH	132.1, CH	
11′/13′	116.4, CH	116.3, CH	116.8, CH	116.2, CH	
12′	156.7, C	157.2, C	157.4, C	163.2, C	
CH_3_N-2	45.8, CH_3_	46.4, CH_3_	46.1, CH_3_	46.3, CH_3_	46.3, CH_3_
CH_3_O-6	56.7, CH_3_	57.2, CH_3_	57.2, CH_3_	57.2, CH_3_	57.2, CH_3_
CH_3_O-7	56.8, CH_3_	57.0, CH_3_	56.9, CH_3_	56.9, CH_3_	56.9, CH_3_
CH_3_O-12	55.7, CH_3_	56.3, CH_3_		56.2, CH_3_	
CH_3_N-2′	45.9, CH_3_	39.9, CH_3_	46.1, CH_3_		
CH_3_O-6′	56.8, CH_3_	55.9, CH_3_	57.2, CH_3_		
CH_3_O-7′	56.6, CH_3_	55.1, CH_3_	56.9, CH_3_		

Compound **2** (Figure 4) was isolated as a bright yellow solid, and the molecular formula for the free base of **2** was determined to be C_39_H_43_N_2_O_6_^+^ by HRESIMS data on the [M – CF_3_COO^–^]^+^ ion at *m*/*z* 635.3108 (calcd for C_39_H_43_N_2_O_6_^+^, 635.3116). In the ^13^C NMR spectrum of **2** (Table 1), 39 carbon signals were observed, assigned as seven methyls, of which five are part of methoxy groups (δ_C_ 55.1, 55.9, 56.3, 57.0, and 57.2), four methylenes, including one bearing a heteroatom (δ_C_ 44.7), one aliphatic methine bearing heteroatoms (δ_C_ 64.3), and 27 aromatic carbons arising from four aromatic rings. The ^1^H NMR spectrum (Table 2) indicated the presence of one symmetrical *para*-disubstituted aromatic ring [δ_H_ 6.77 (2H, d, *J* = 8.7 Hz), 7.06 (2H, d, *J* = 8.7 Hz)], a 1,2,4-trisubstituted aromatic ring [δ_H_ 6.95 (d, *J* = 2.4 Hz), 7.11 (d, *J* = 8.9 Hz), 6.92 (dd, *J* = 2.4, 8.9 Hz)], a 1,6,7-trisubstituted isoquinolinium moiety [δ_H_ 8.53 (d, *J* = 7.2 Hz), 8.21 (d, *J* = 7.2 Hz), 7.73 (s), 7.82 (s)], two aromatic proton singlets [δ_H_ 6.85, 5.71 (each 1H, s)] due to a 1,2,4,5-tetrasubstituted aromatic ring, and seven heteroatom-bearing methyl singlets [δ_H_ 2.84, 3.29, 3.69, 3.74, 3.93, 4.04, 4.30]. These NMR spectroscopic features were closely related to those of phaeantharine (**1**). However, instead of the second unit of a 1,6,7-trisubstituted isoquinolinium moiety in **1**, compound **2** showed a 1,2,4,5-tetrasubstituted aromatic ring, together with additional aliphatic methylenes (δ_C_ 21.3, 44.7) and methine (δ_C_ 64.3) carbons, suggesting an aliphatic B′-ring.

The structures of the A, A′, B, C, and C′ aromatic rings and their connections in compound **2** were determined by comparison to the observed data of phaeantharine (**1**). The structure of the B′-ring of **2** was determined from the HMBC correlations of H-1′ (δ_H_ 4.53, m, CH) with C-8′a (δ_C_ 121.3, C), H_2_-3′ (δ_H_ 3.73, 3.39, m, CH_2_) with C-1′/C-4’a, H_2_-4′ (δ_H_ 2.99, m, CH_2_) with C-4′a/C-8′a, and the CH_3_N (δ_H_ 2.84) group with δ_C_ 64.3 (CH, C-1′) and 44.7 (CH_2_, C-3′). Peaks of the aromatic proton at δ_H_ 6.77 (2H, H-11′, H-13′) with C-9′ (δ_C_ 130.1)/C-12′ (δ_C_ 157.2), 7.06 (2H, H-10′, H-14′) with C-12′/C-α′ (δ_C_ 40.2), both H_2_-α′ (δ_H_ 3.37, 2.96, m, CH_2_) and H-1′ (δ_H_ 4.53) with C-8′a, and H-α*′* with C-14′ (δ_C_ 131.7) confirmed the connection of the *para*-disubstituted aromatic C′-ring via another methylene group, C-α*′*, to the heteroatom-bearing methine (δ_C_ 64.3, C-1′), which was connected to C-8′a of the A′-ring. HMBC correlations of the methoxy protons (δ_H_ 4.04, s, CH_3_O-6, 3.93, s, CH_3_O-7, 3.69, s, CH_3_O-12, 3.74, s, CH_3_O-6′, and 3.29, s, CH_3_O-7′) with deshielded C-6 (δ_C_ 157.2), C-7 (δ_C_ 153.2), C-12 (δ_C_ 151.0), C-6′ (δ_C_ 148.9), and C-7′ (δ_C_ 146.9), respectively, provided the 2D structure of compound **2**.

The absolute configuration of the C-1′ stereogenic center of **2** was established by the electronic circular dichroism (ECD) spectrum, which exhibited Cotton effects similar to the calculated spectrum of the 1′*R*-**2** diastereoisomer (Figure 6). On the basis of the above evidence, the structure of compound **2** was elucidated as a new compound, named (*R*)-nomimantharine trifluoroacetate.

**Figure 5:**
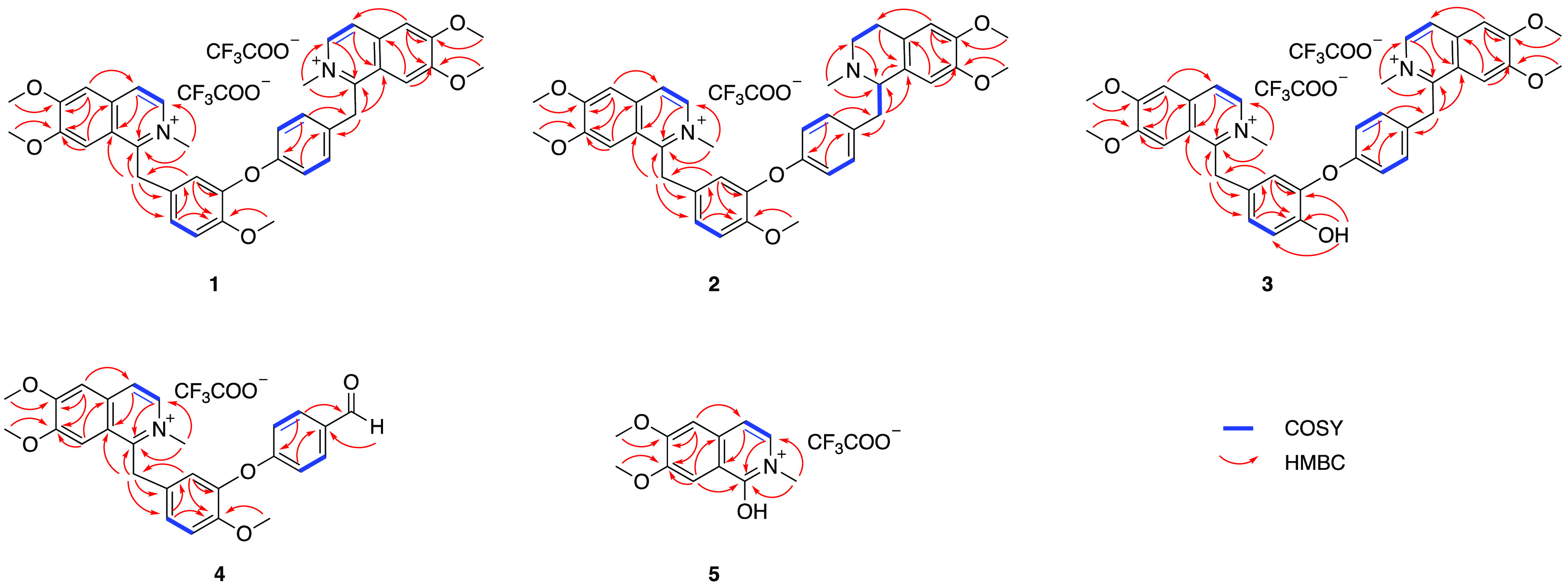
COSY and key HMBC correlations of compounds **1**–**5**.

**Figure 6:**
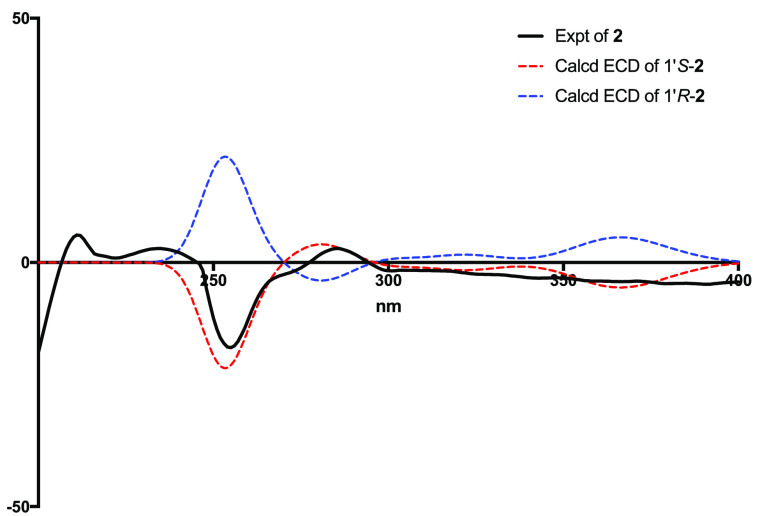
Experimental and computed ECD spectra of **2**.

Compound **3** (Figure 4) was isolated as a bright yellow solid, and the HRESIMS data showed a molecular ion at *m*/*z* 617.2641 [M – 2CF_3_COO^–^ – H]^+^ (calcd for C_38_H_37_N_2_O_6_^+^, 617.2646), which was consistent with a molecular formula for the free base of **3** as C_38_H_38_N_2_O_6_^2+^, 14 Da less than that of **1**. The UV spectrum was similar to those of **1** and **2**. A comparison of the ^1^H and ^13^C NMR spectroscopic data (Table 1) with those of **1** showed that the only difference was the absence of one methoxy group in the C-ring of **3**. In the ^1^H NMR spectrum of **3**, four methoxy proton singlets were observed (δ_H_ 4.06, CH_3_O-6, 3.88, CH_3_O-7, 4.07, CH_3_O-6′, 3.96, CH_3_O-7′) with a phenolic proton at δ_H_ 9.73. HMBC correlations (Figure 5) from δ_H_ 9.73 to C-11 (δ_C_ 142.6, C)/C-12(δ_C_ 149.0, C)/C-13(δ_C_ 118.1, CH) supported the connection of the hydroxy group to C-12. Finally, compound **3** was identified as a new compound, named 12-de-*O*-methylphaeantharine trifluoroacetate.

Compound **4** (Figure 4) was isolated as a yellow solid, and the HRESIMS data showed a molecular ion at *m*/*z* 444.1800 [M – CF_3_COO^–^]^+^ (calcd for C_27_H_26_NO_5_^+^, 444.1805), which was consistent with a molecular formula for the free base of **4** as C_27_H_26_NO_5_^+^. Analysis of its ^1^H and ^13^C NMR spectroscopic data suggested that instead of having two symmetrical units as compounds **1**–**3**, there was only one benzyltetrahydroisoquinoline unit present in compound **4**. This was consistent with the molecular formula, accommodating one nitrogen atom. The ^1^H NMR and ^13^C NMR spectroscopic data of rings A, B, C, and C′ were identical to those of **1** and **2** (Tables 1, 2). A significantly deshielded chemical shift at δ_C_ 191.6 was observed in **4**, suggesting a formyl proton resonating at δ_H_ 9.89 by HSQC data. The formyl group was assigned at C-α*′* due to the HMBC correlations from δ_H_ 9.89 to C-10′/C-14′ (δ_C_ 132.1, CH) and from H-10′/H-14′ (δ_H_ 7.86, d, *J* = 8.7 Hz) to δ_C_ 191.6. Therefore, compound **4** was determined to be nominanthranal trifluoroacetate.

Compound **5** (Figure 4) was obtained as a white solid and gave a molecular formula for the free base as C_12_H_14_NO_3_^+^, as deduced from the positive HRESIMS (*m*/*z* 220.0966 [M – CF_3_COO^–^]^+^, calcd for C_12_H_14_NO_3_^+^, 220.0968). The ^1^H and ^13^C NMR data of **5** suggested it possessed a similar 6,7-dimethoxy-2-methylisoquinolinium moiety to compounds **1**–**4**. The detected nine aromatic carbons were assigned to the isoquinolinium moiety based on comparison with data of compounds **1**–**4**. Three heteroatom-bearing methyl singlets (δ_H_ 4.32, 4.07, 3.94) were assigned to CH_3_N-2, CH_3_O-6, and CH_3_O-7 on the basis of their HMBC correlations with C-1 (δ_C_ 161.2, C)/C-3 (δ_C_ 132.4, CH), C-6 (δ_C_ 153.3, C), and C-7 (δ_C_ 149.2, C), respectively. Comparison of the molecular formula suggested the presence of a hydroxy group, which was detected as a broad signal at δ_H_ 6.54. The deshielded chemical shift of C-1 (δ_C_ 161.2, C) suggested its connection with the hydroxy group. Consequently, **5** was deduced as the trifluoroacetate salt of the enolic form of *N*-methyl-6,7-dimethoxyisoquinolone (**6**).^19^

Compounds **1**–**5** were subjected to in vitro screening against *Mycobacteria* strains using the *M. smegmatis* strain mc^2^155, *M. tuberculosis* mc^2^ 6220 biosafety level 2 (BSL2) strain, and the wild-type strain H37Rv (Table 3). (*R*)-Nomimantharine trifluoroacetate (**2**) showed moderate activities against the *M. smegmatis* strain mc^2^155 and *Mtb* mc^2^ 6220 BSL2 strain with MIC values of 39.4 and 12.5 μM, respectively. Phaeantharine trifluoroacetate (**1**) displayed weak activity against the *M. smegmatis* strain mc^2^155, but no inhibition was observed in the *Mtb* screens. Compounds **3**–**5** showed no activity (MIC > 100 μg/mL) against the *M. smegmatis* strain mc^2^155 and were, therefore, not evaluated in *Mtb* assays. Comparisons of the MIC values obtained for these compounds suggested that saturation of the B′-ring increases antitubercular activity.

**Table 3:** In Vitro Antimicrobial Activity of the Isolated Compounds

strain		**1**	**2**	**3**	**4**	**5**
*M. smegmatis* MIC (μM)		158.2	39.4	>100	>100	>100
*Mtb* mc^2^ 6220 BSL2 growth inhibition (%)		0	70 at 6.25 μM	NT^a^	NT	NT
*Mtb* mc^2^ 6220 BSL2MIC (μM)		NT	12.5	NT	NT	NT
*Mtb* H37Rv MIC (μM)		NT	>100	NT	NT	NT
*E. coli* MIC (μM)		39.6	157.5	>100	>100	>100
*Staphylococcus aureus* MIC (μM)		9.9	39.4	>100	>100	>100
MRSA MIC (μM)	strain 1	9.9	39.4	NT	NT	NT
	strain 2	39.6	39.4	NT	NT	NT
	strain 3	39.6	19.7	NT	NT	NT
	strain 4	9.9	39.4	NT	NT	NT
	strain 5	9.9	19.7	NT	NT	NT

a NT: not tested.

Compounds **1**–**5** were evaluated for antimicrobial activity against *E. coli* and *S. aureus*. Phaeantharine trifluoroacetate (**1**) exhibited promising activity against *E. coli* and *S. aureus* with MIC values of 39.6 and 9.9 μM, respectively. (*R*)-Nomimantharine trifluoroacetate (**2**) showed weak activity in both assays with MIC values of 157.5 and 39.4 μM, respectively, whereas compounds **3**–**5** were inactive (MIC > 100 μg/mL). In order to further evaluate the antibacterial potential of phaeantharine trifluoroacetate (**1**) and (*R*)-nomimantharine trifluoroacetate (**2**) against drug-resistant *S. aureus* strains, their activities were measured against five clinical isolates of oxacillin/methicillin-resistant *S. aureus* (MRSA) (Table 3). Both compounds showed similar or even stronger levels of activity against the resistant isolates, indicating their potential as anti-MRSA agents.

## Experimental Section

### General Experimental Procedures

Optical rotations were recorded on a JASCO P-1020 polarimeter (10 cm cell). NMR spectra were recorded in DMSO-*d*_6_ (δ_H_ 2.50 and δ_C_ 39.5) at 25 °C on a Bruker Avance HDX 800 MHz spectrometer equipped with a TCI cryoprobe. HRMS data were recorded on a Bruker maXis II ETD ESI-qTOF. An Edwards Instrument Company Bioline orbital shaker was used for extraction. The HPLC system for fractionation for initial *Mtb* screening was a Waters 600 pump (Milford, MA, USA) fitted with a 996-photodiode array detector (PDA) and Gilson FC204 fraction collector (Middleton, WI, USA). Semipreparative HPLC was performed on a Thermo Ultimate 3000 with a PDA detector. A Phenomenex C_18_ Monolithic column (5 μm, 4.6 × 100 mm) was used for analytical HPLC; two Thermo Hypersil Gold C_18_ columns (5 μm, 21.2 × 250 mm and 5 μm, 10 × 250 mm) were used for semipreparative HPLC. All solvents used for extraction, chromatography, [α]_D_, and MS were HPLC grade, and H_2_O was Millipore Milli-Q PF filtered.

### Plant Material

The sample of *D. aromatica* NB024954 was collected and identified by P.I.F. in April 1999 from East of Rossville in north Queensland. A voucher specimen is lodged in the Queensland Herbarium (BRI-AQ606149).

### Extraction and Purification of Compounds **1**–**5**

The ground and freeze-dried *D. aromatica* (17 g) was extracted with 95% ethanol (3 × 300 mL) overnight at room temperature (rt) to afford a crude extract (2.75 g). The crude extract was fractionated using a Sephadex LH-20 column. Five fractions (I–V) were collected by eluting with 100% MeOH. Fraction II, containing the alkaloids, was chromatographed by HPLC (gradient from 60% H_2_O/40% MeOH with 0.1% TFA to 20% H_2_O/80% MeOH with 0.1% TFA, 9 mL/min) using a semipreparative reversed-phase Thermo Hypersil Gold C_18_ column (5 μm, 21.2 × 250 mm), leading to the isolation of phaeantharine trifluoroacetate (**1**) (retention time: 16.5 min, 12.6 mg), (*R*)-nomimantharine trifluoroacetate (**2**) (retention time: 15.6 min, 5.7 mg), and four subfractions, A–D. Subfraction B was further chromatographed by HPLC (isocratic 27% H_2_O/MeOH with 0.1% TFA, 4 mL/min) using a semipreparative reversed-phase Thermo Hypersil Gold C_18_ column (5 μm, 10 × 250 mm), leading to the isolation of 12-de-*O*-methylphaeantharine trifluoroacetate (**3**) (retention time: 98.8 min, 1.2 mg). Subfraction C was chromatographed by the same Thermo Hypersil Gold C_18_ column (5 μm, 10 × 250 mm) eluting at a flow rate of 4 mL/min with 35% H_2_O/MeOH with 0.1% TFA to yield nominanthranal trifluoroacetate (**4**) (retention time: 44.9 min, 2.1 mg) and the enolic form of 1-hydroxy-6,7-dimethoxy-2-methylisoquinoline trifluoroacetate (**5**) (retention time: 41.2 min, 1.7 mg).

#### (*R*)-Nomimantharine trifluoroacetate (**2**):

bright yellow solid;  −147 (*c* 0.03, MeOH); UV (MeOH) λ_max_ (log ε) 209 nm (3.42), 228 nm (2.67), 256 nm (3.39), 282 nm (0.63), 316 nm (0.62); ^1^H NMR (800 MHz, DMSO-*d*_6_) and ^13^C NMR data (200 MHz, DMSO-*d*_6_), Tables 1 and 2; (+)-HRESIMS *m*/*z* 635.3108 [M – CF_3_COO^–^]^+^ (calcd for C_39_H_43_N_2_O_6_^+^, 635.3116).

#### 12-De-*O*-methylphaeantharine trifluoroacetate (**3**):

bright yellow solid; UV (MeOH) λ_max_ (log ε) 199 nm (2.60), 255 nm (2.92), 316 nm (0.53); ^1^H NMR (800 MHz, DMSO-*d*_6_) and ^13^C NMR data (200 MHz, DMSO-*d*_6_), Tables 1 and 2; (+)-HRESIMS *m*/*z* 309.1357 [M – 2CF_3_COO^–^]^2+^ (calcd for C_38_H_38_N_2_O_6_^2+^, 309.1359), 617.2641 [M – 2CF_3_COO^–^ – H]^+^ (calcd for C_38_H_37_N_2_O_6_^+^, 617.2646).

#### Nominanthranal trifluoroacetate (**4**):

yellow solid; UV (MeOH) λ_max_ (log ε) 204 nm (3.20), 227 nm (1.76), 256 nm (2.36); ^1^H NMR (800 MHz, DMSO-*d*_6_) and ^13^C NMR data (200 MHz, DMSO-*d*_6_), Tables 1 and 2; (+)-HRESIMS *m*/*z* 444.1800 [M – CF_3_COO^–^]^+^ (calcd for C_27_H_26_NO_5_^+^, 444.1805).

#### 1-Hydroxy-6,7-dimethoxy-2-methylisoquinoline trifluoroacetate (**5**):

white solid; UV (MeOH) λ_max_ (log ε) 205 nm (1.43), 226 nm (0.98), 255 nm (2.05); ^1^H NMR (800 MHz, DMSO-*d*_6_) and ^13^C NMR data (200 MHz, DMSO-*d*_6_), Tables 1 and 2; (+)-HRESIMS *m*/*z* 220.0966 [M – 2CF_3_COO^–^]^+^ (calcd for C_12_H_14_NO_3_^+^, 220.0968).

### DFT Calculations

The calculations were performed by using the density functional theory (DFT) as carried out in the Gaussian 03 program. The preliminary conformational distribution search was performed by HyperChem Release 8.0 software. GaussView 5.0 was used to view the conformational structures and change the input file for calculation. All ground-state geometries were optimized at the B3LYP/6-31G(d) level. Conformers within a 2 kcal/mol energy threshold from the global minimum were selected to calculate the electronic transitions. The overall calculated ECD spectra were obtained according to the Boltzmann weighting of each conformer.

### Biological Assays

Compounds were evaluated for their antimycobacterial activities against *M. smegmatis* strain mc^2^155 (ATCC 70084),^20^*M. tuberculosis* mc^2^ 6220 biosafety level 2 (BSL2) strain, and *M. tuberculosis* H37Rv.^21^ All the experiments were performed in triplicate.

Antimicrobial assays were performed according to the previously reported method.^22^

## Supporting Information Available

Spectroscopic data of phaeantharine trifluoroacetate (**1**), 1D and 2D NMR and MS data of compounds **1**–**5**, stable conformers of *R*-nomimantharine trifluoroacetate (**2**) (PDF)

## Dedication

Dedicated to Dr. A. Douglas Kinghorn, The Ohio State University, for his pioneering work on bioactive natural products.
