# Supplementary material for: Antimicrobial Benzyltetrahydroisoquinoline-Derived Alkaloids from the Leaves of Doryphora aromatica
Source: J Nat Prod. 2021 Mar 5;84(3):676–82. doi: 10.1021/acs.jnatprod.0c01093 (PMC8008450; doi:10.1021/acs.jnatprod.0c01093)
Supplement: Supplementary file 1 — np0c01093_si_001.pdf [file np0c01093_si_001.pdf]

Supporting Information

# Antimicrobial Benzyltetrahydroisoquinoline-Derived Alkaloids from the Leaves of *Doryphora aromatica*

Miaomiao Liu,[†,*] Jianying Han,[†] Yunjiang Feng,[†] Gordon Guymer,[¶] Paul I. Forster,[¶] and Ronald J Quinn[†,*]

[†] Griffith Institute for Drug Discovery, Griffith University, Brisbane, QLD 4111, Australia

[¶] Queensland Herbarium, Department of Environment and Science, Brisbane Botanic Gardens, Brisbane, QLD 4066, Australia

**Contents**

Spectroscopic data of phaeantharine trifluoroacetate (**1**)

**Figure S1**. $^1$H NMR spectrum of phaeantharine trifluoroacetate (**1**)

**Figure S2**. $^{13}$C NMR spectrum of phaeantharine trifluoroacetate (**1**)

**Figure S3**. COSY NMR spectrum of phaeantharine trifluoroacetate (**1**)

**Figure S4**. HSQC NMR spectrum of phaeantharine trifluoroacetate (**1**)

**Figure S5**. HMBC NMR spectrum of phaeantharine trifluoroacetate (**1**)

**Figure S6**. HRMS spectrum of phaeantharine trifluoroacetate (**1**)

**Figure S7**. $^1$H NMR spectrum of (*R*)-nomimantharine trifluoroacetate (**2**)

**Figure S8**. $^{13}$C NMR spectrum of (*R*)-nomimantharine trifluoroacetate (**2**)

**Figure S9**. COSY NMR spectrum of (*R)*-nomimantharine trifluoroacetate (**2**)

**Figure S10**. HSQC NMR spectrum of (*R*)-nomimantharine trifluoroacetate (**2**)

**Figure S11**. HMBC NMR spectrum of (*R*)-nomimantharine trifluoroacetate (**2**)

**Figure S12**. HRMS spectrum of (*R*)-nomimantharine trifluoroacetate (**2**)

**Figure S13**. $^1$H NMR spectrum of 12-de-*O*-methyphaeantharine trifluoroacetate (**3**)

**Figure S14**. COSY NMR spectrum of 12-de-*O*-methyphaeantharine trifluoroacetate (**3**)

**Figure S15**. HSQC NMR spectrum of 12-de-*O*-methyphaeantharine trifluoroacetate (**3**)

**Figure S16**. HMBC NMR spectrum of 12-de-*O*-methyphaeantharine trifluoroacetate (**3**)

**Figure S17**. HRMS spectrum of 12-de-*O*-methyphaeantharine trifluoroacetate (**3**)

**Figure S18**. $^1$H NMR spectrum of nominanthranal trifluoroacetate (**4**)

**Figure S19**. COSY NMR spectrum of nominanthranal trifluoroacetate (**4**)

**Figure S20**. HSQC NMR spectrum of nominanthranal trifluoroacetate (**4**)

**Figure S21**. HMBC NMR spectrum of nominanthranal trifluoroacetate (**4**)

**Figure S22**. HRMS spectrum of nominanthranal trifluoroacetate (**4**)

**Figure S23**. $^{1}$H NMR spectrum of 1-hydroxyl-6,7-dimethoxy-2-methylisoquinoline trifluoroacetate (**5**)

**Figure S24**. COSY NMR spectrum of 1-hydroxyl-6,7-dimethoxy-2-methylisoquinoline trifluoroacetate (**5**)

**Figure S25**. HSQC NMR spectrum of 1-hydroxyl-6,7-dimethoxy-2-methylisoquinoline trifluoroacetate (**5**)

**Figure S26**. HMBC NMR spectrum of 1-hydroxyl-6,7-dimethoxy-2-methylisoquinoline trifluoroacetate (**5**)

**Figure S27**. HRMS spectrum of 1-hydroxyl-6,7-dimethoxy-2-methylisoquinoline trifluoroacetate (**5**)

Table S1. Stable conformers of (*R*)-nomimantharine trifluoroacetate (**2**)

Spectroscopic data of phaeantharine trifluoroacetate (**1**)

*Phaeantharine trifluoroacetate (1):* bright yellow solid; UV (MeOH) $\lambda_{max}$ (log $\varepsilon$) 199 nm (1.43), 229 nm (2.24), 256 nm (4.08), 316 nm (0.85); $^{1}$H NMR (800 MHz, DMSO-$d_6$) and $^{13}$C NMR data (200 MHz, DMSO-$d_6$), Tables 1 and 2; (+)-HRESIMS *m/z* 316.143891 [M − $2CF_3COO^-]^{2+}$ (calcd for $C_{39}H_{40}N_2O_6^{2+}$, 316.143770), *m/z* 631.2813 [M − $2CF_3COO^-$ − H]$^+$ (calcd for $C_{39}H_{39}N_2O_6^+$, 631.2802).

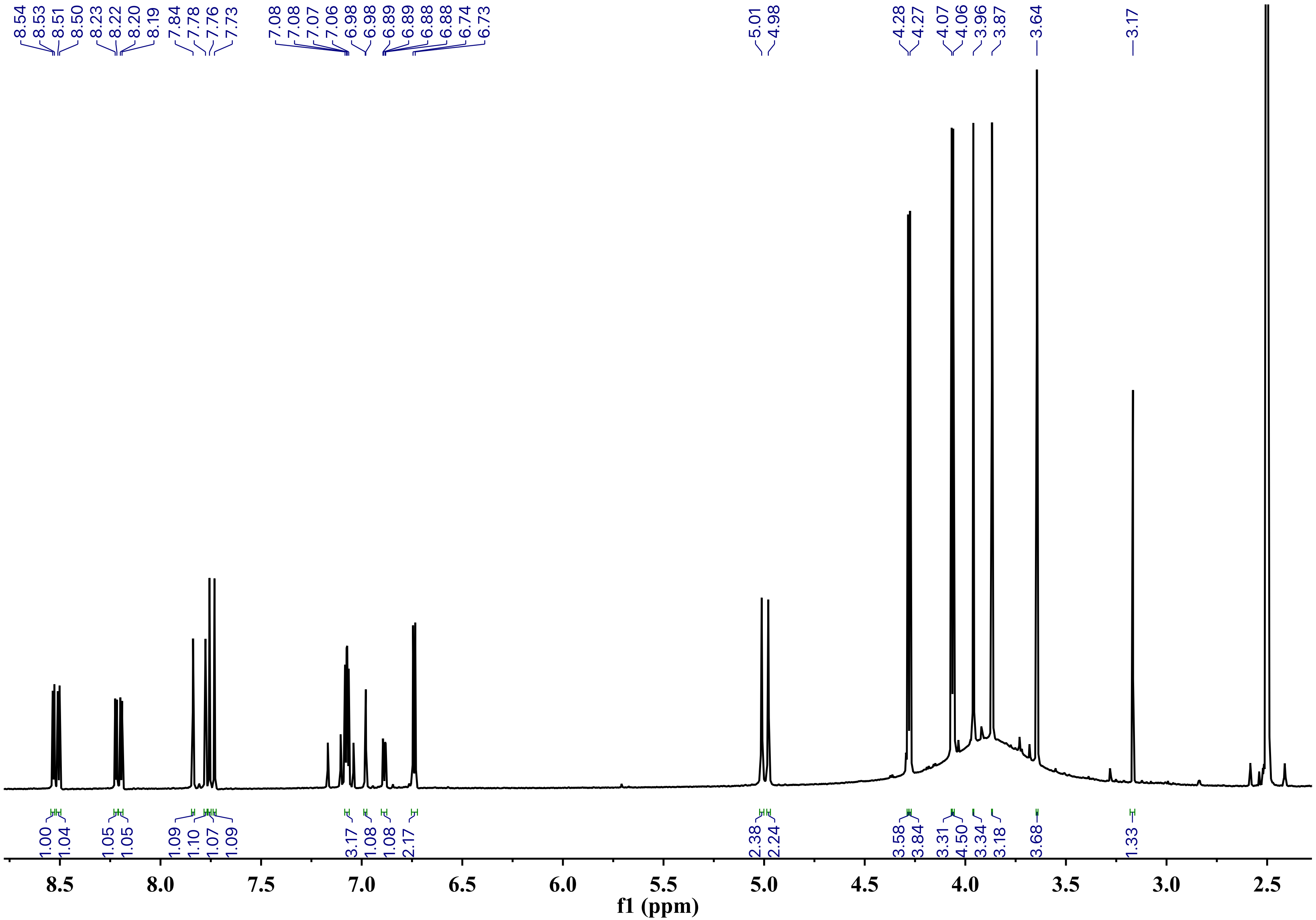


**Figure S1**. $^1$H NMR spectrum of phaeantharine trifluoroacetate (**1**)

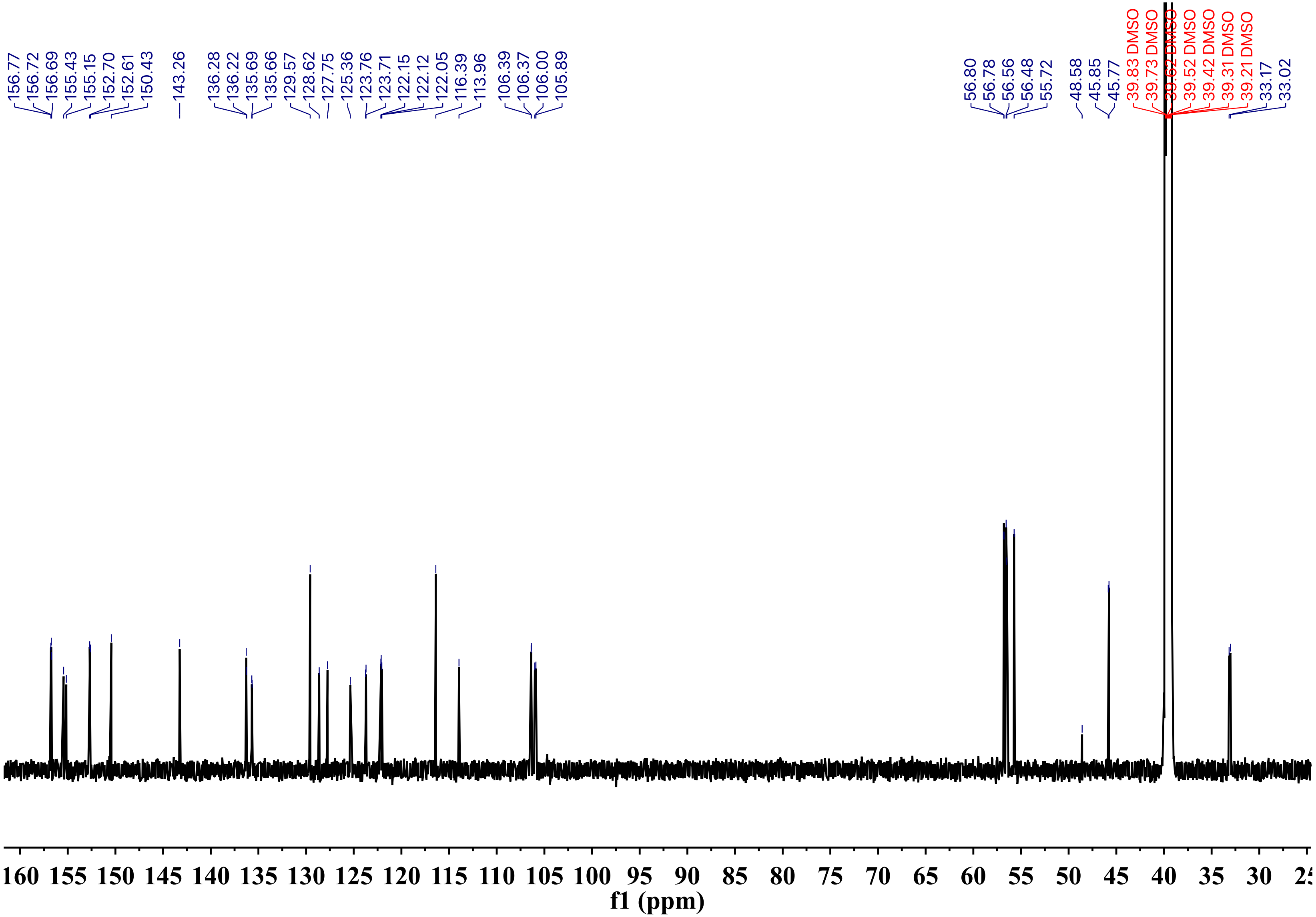


**Figure S2**. $^{13}$C NMR spectrum of phaeantharine trifluoroacetate (**1**)

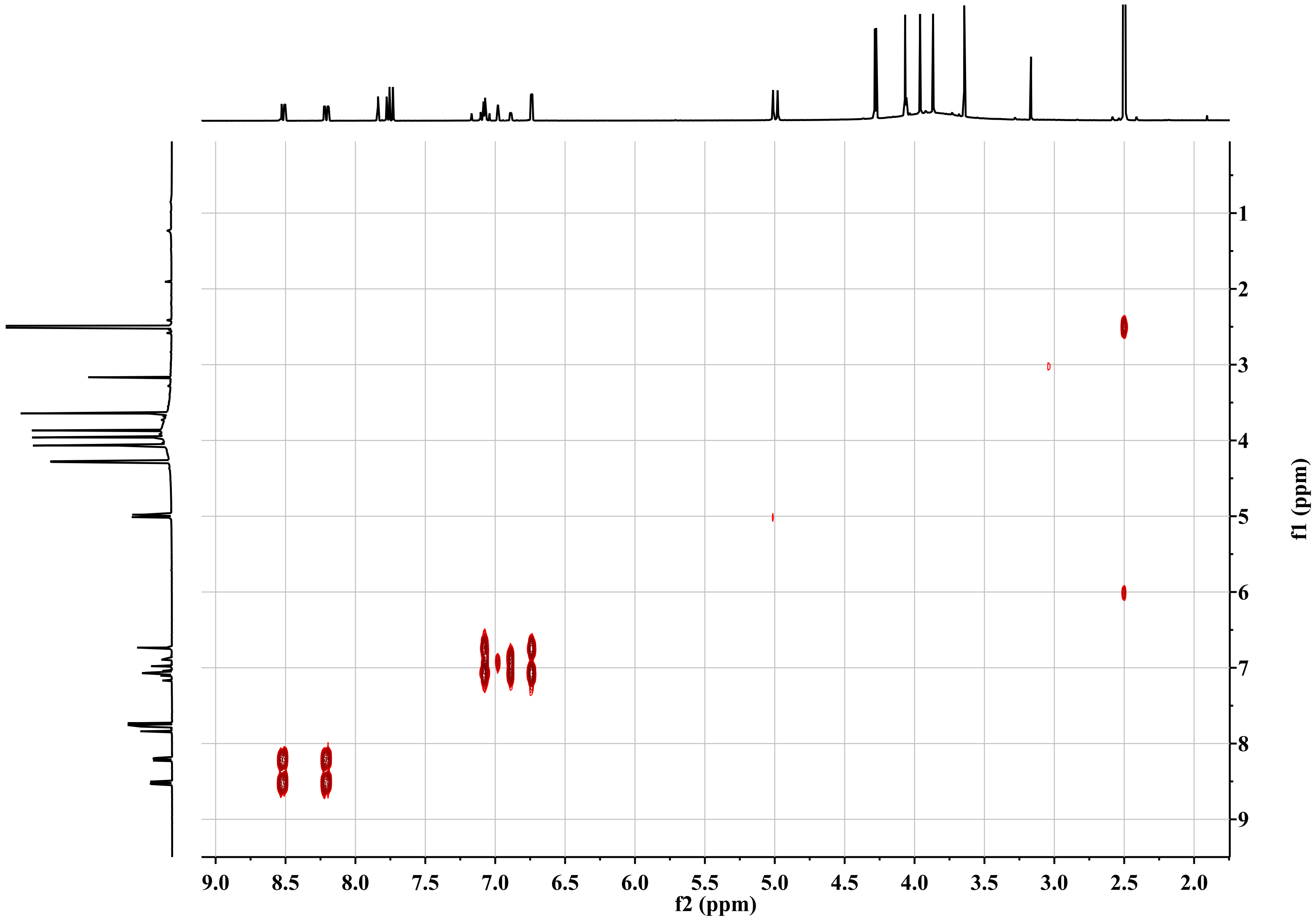


**Figure S3**. COSY NMR spectrum of phaeantharine trifluoroacetate (**1**)

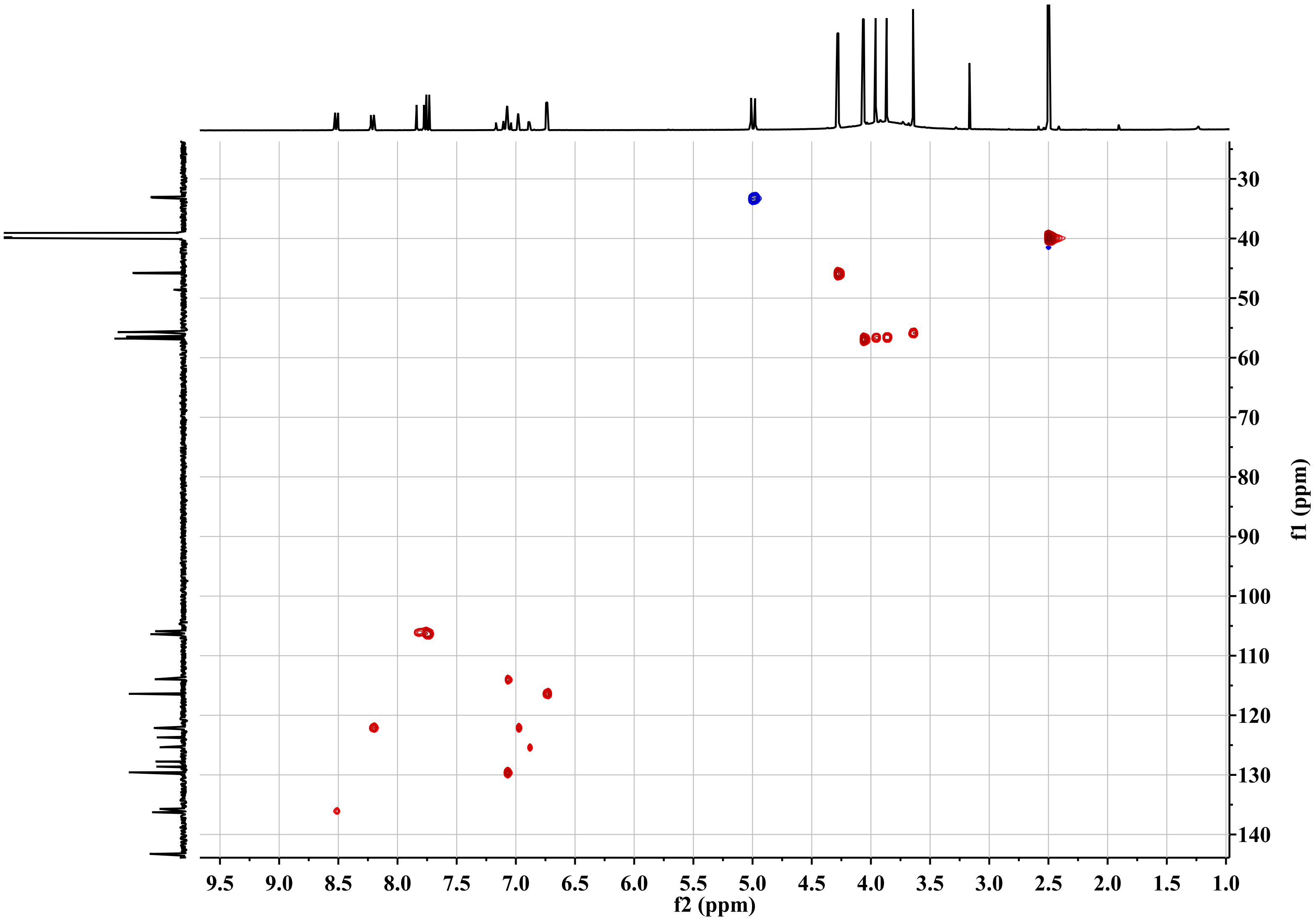


**Figure S4**. HSQC NMR spectrum of phaeantharine trifluoroacetate (**1**)

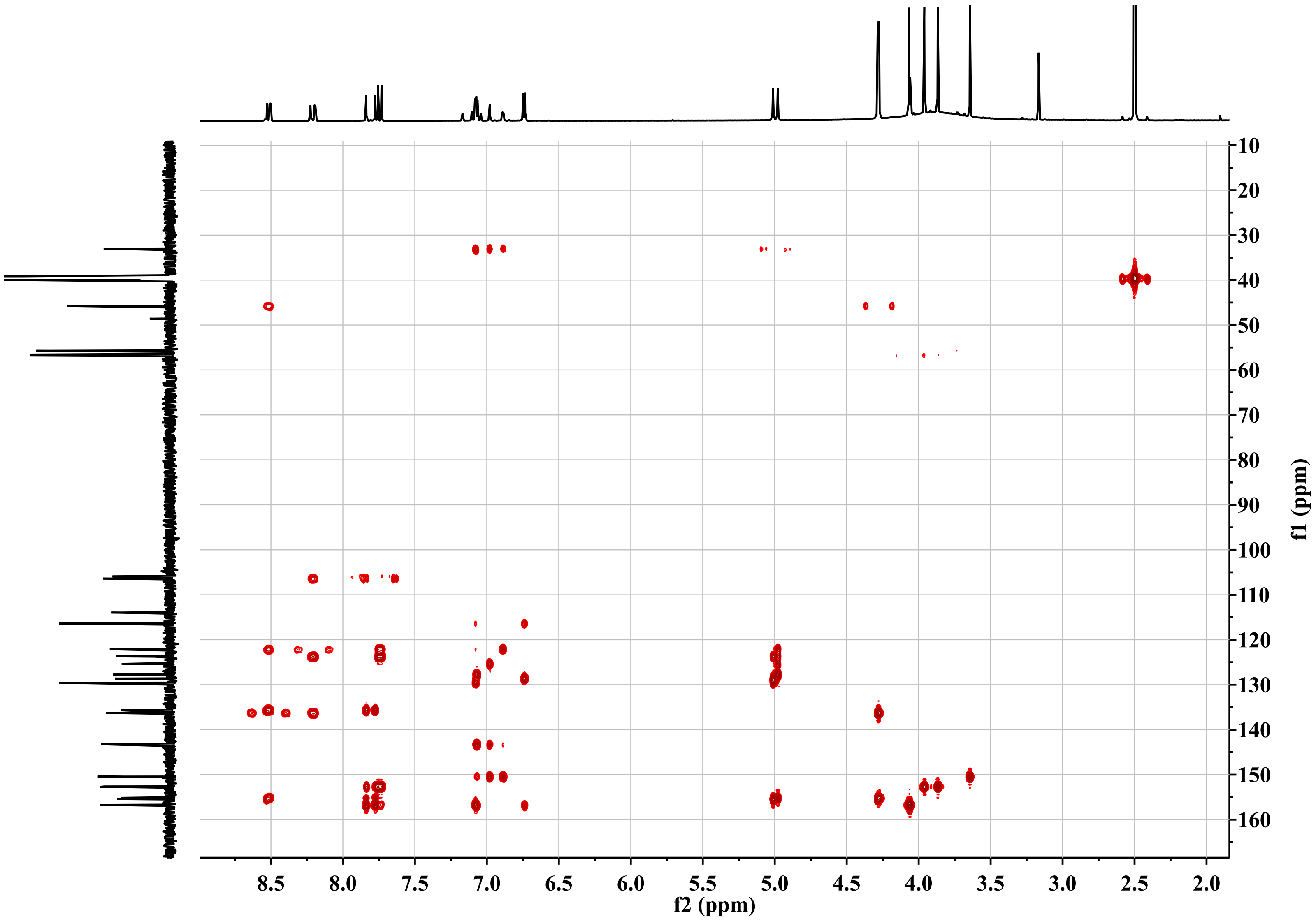


**Figure S5**. HMBC NMR spectrum of phaeantharine trifluoroacetate (**1**)

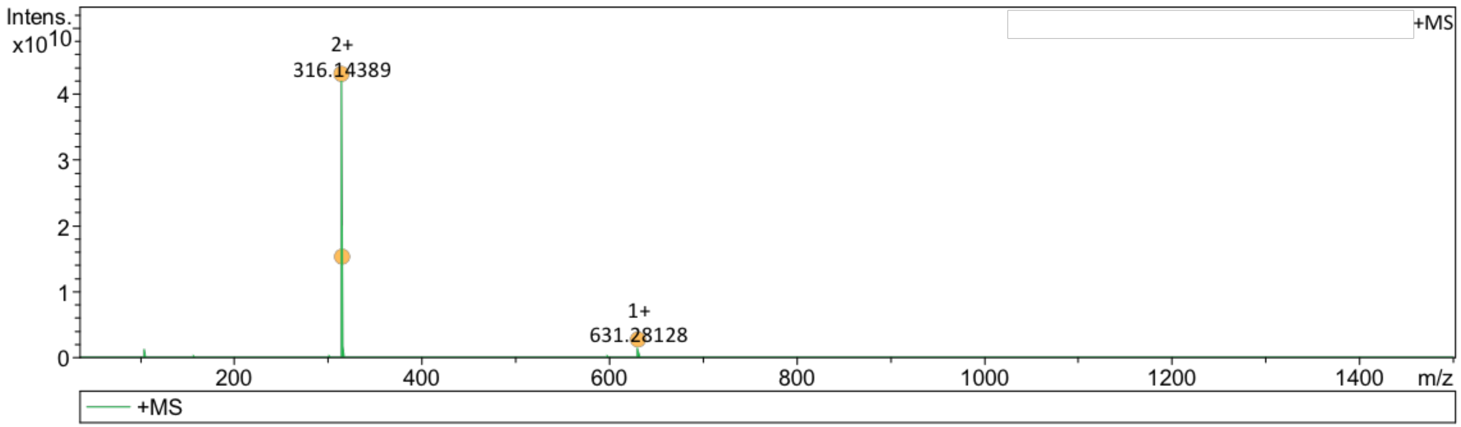


| Means. m/z | Ion formula | calc. m/z | err [ppm] | rdb | e⁻Conf | N-Rule |
|---|---|---|---|---|---|---|
| 316.143891 | $C_{39}H_{40}N_2O_6$ | 316.143770 | -0.4 | 22.0 | even | ok |
| 631.281278 | $C_{39}H_{39}N_2O_6$ | 631.280263 | -1.6 | 22.0 | even | ok |

**Figure S6**. HRMS spectrum of trifluoroacetate phaeantharine (**1**)

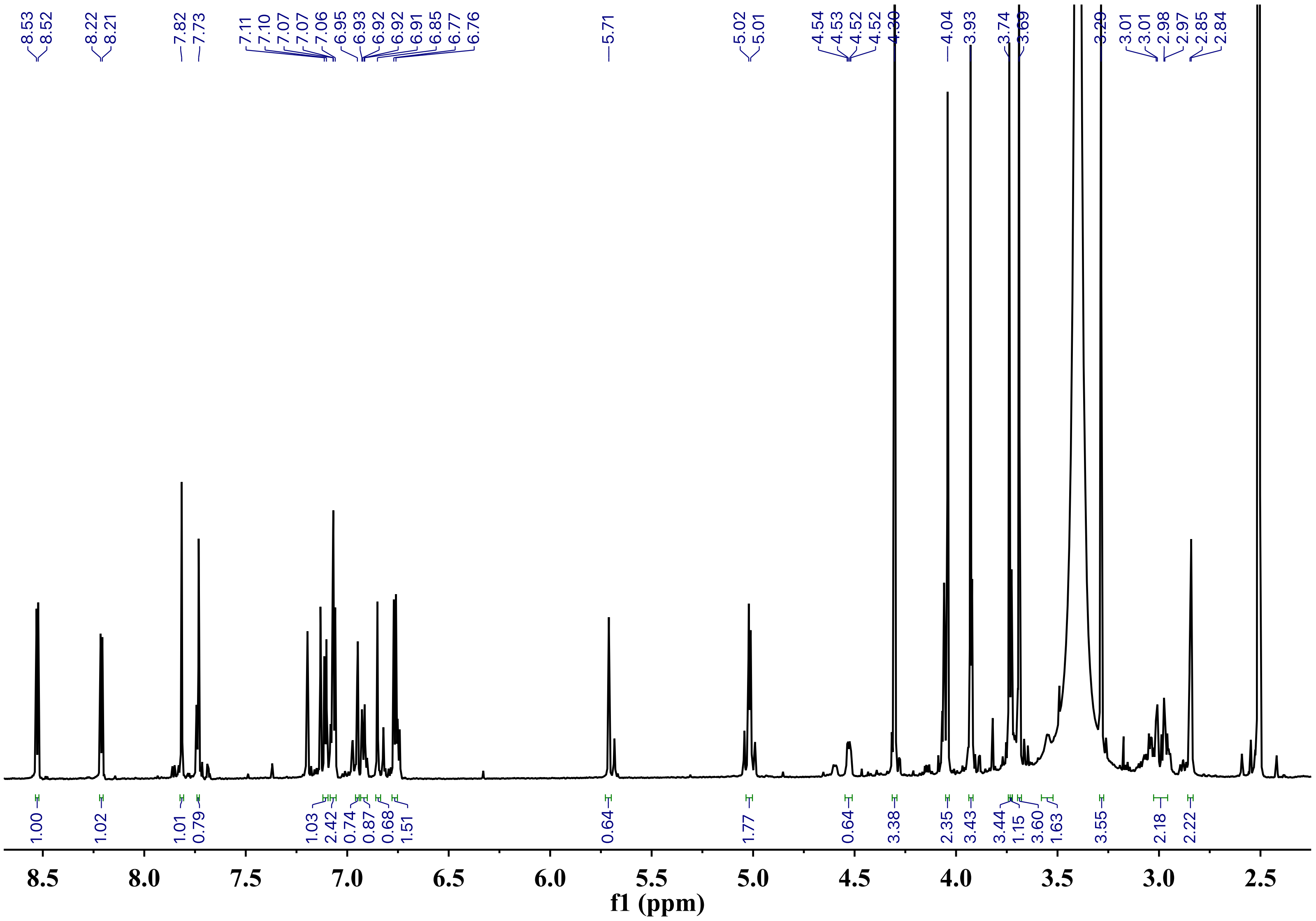


**Figure S7**. $^{1}$H NMR spectrum of (*R*)-nomimantharine trifluoroacetate (**2**)

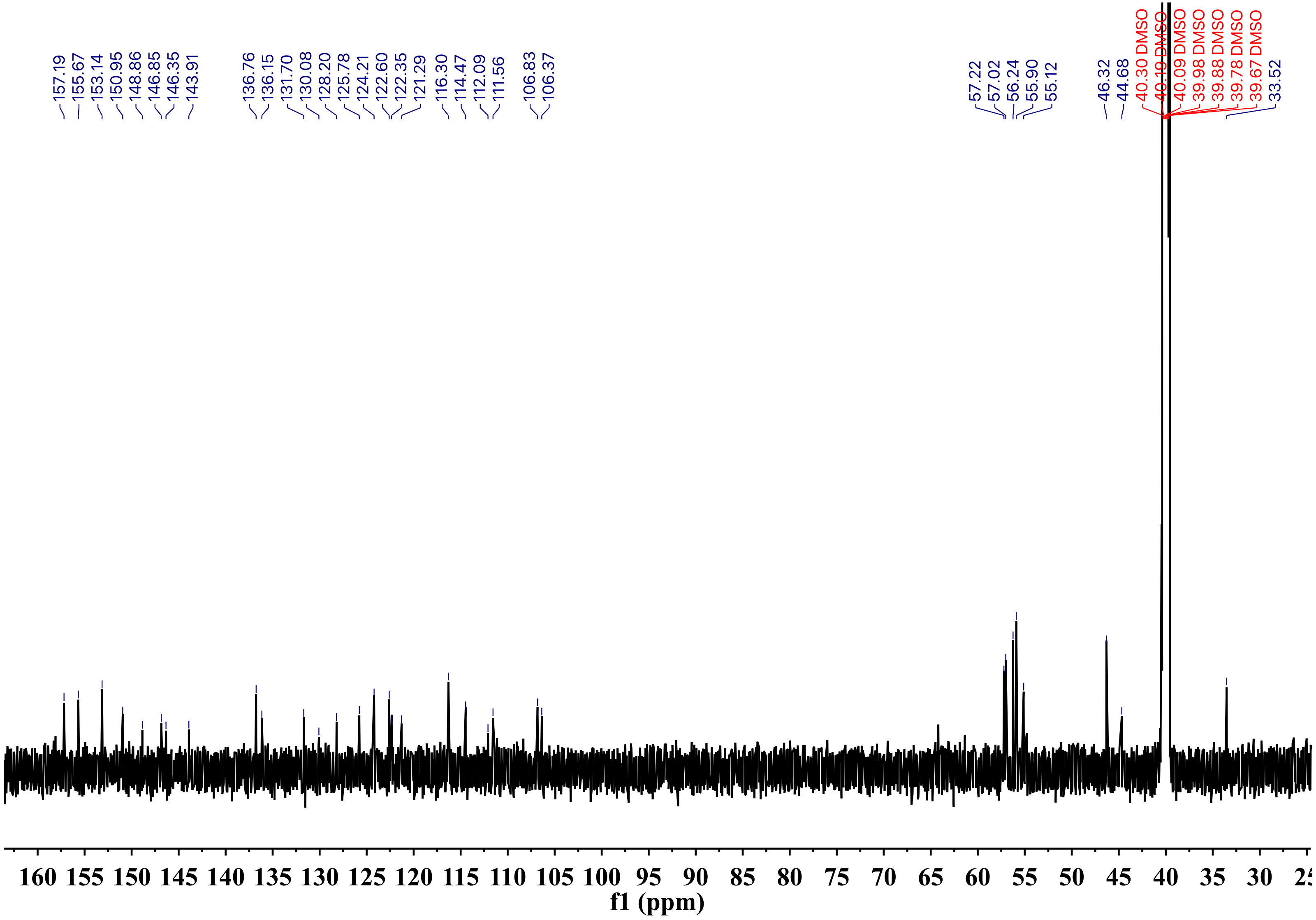


**Figure S8**. $^{13}$C NMR spectrum of (*R*)-nomimantharine trifluoroacetate (**2**)

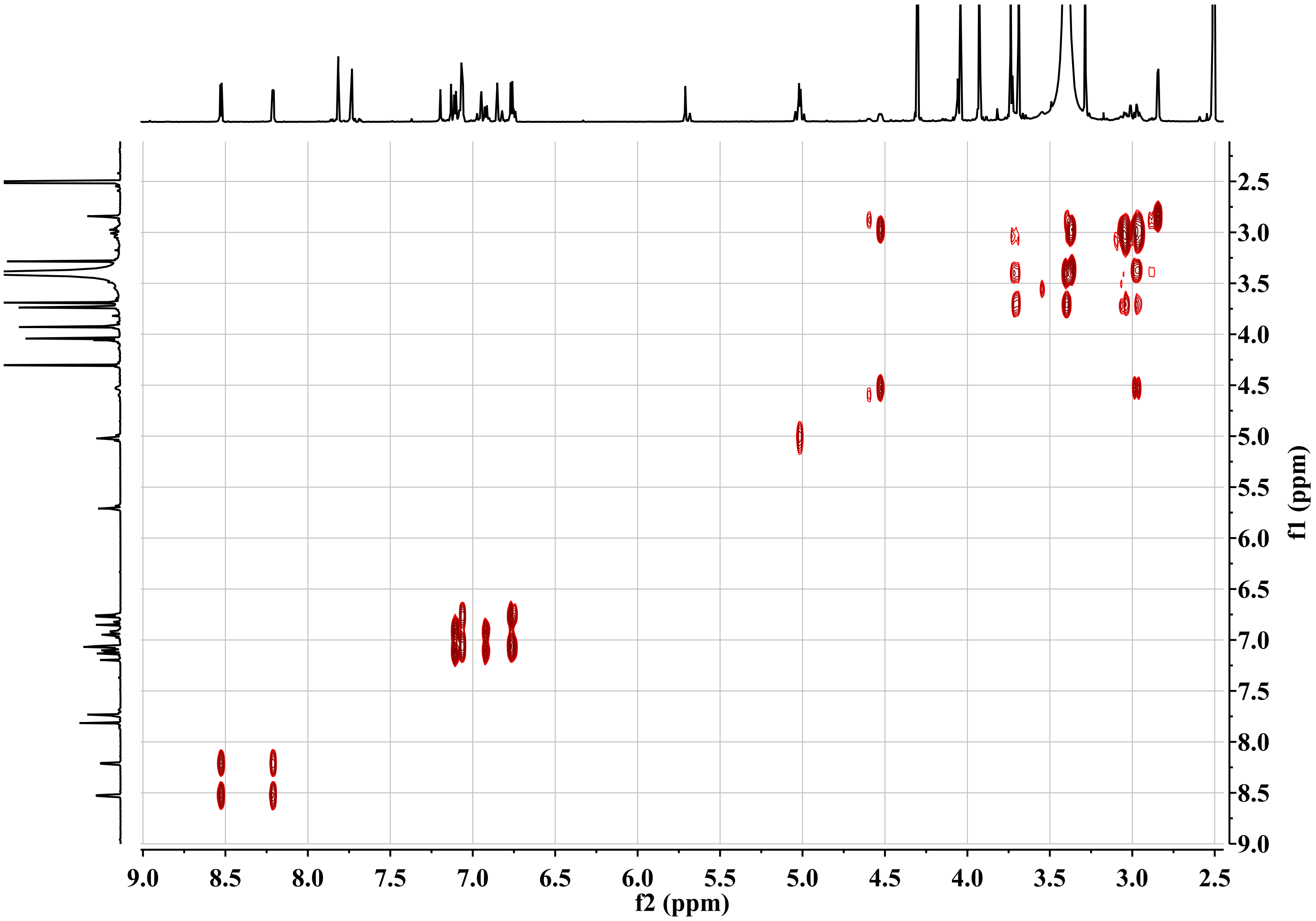


**Figure S9**. COSY NMR spectrum of (*R*)-nomimantharine trifluoroacetate (**2**)

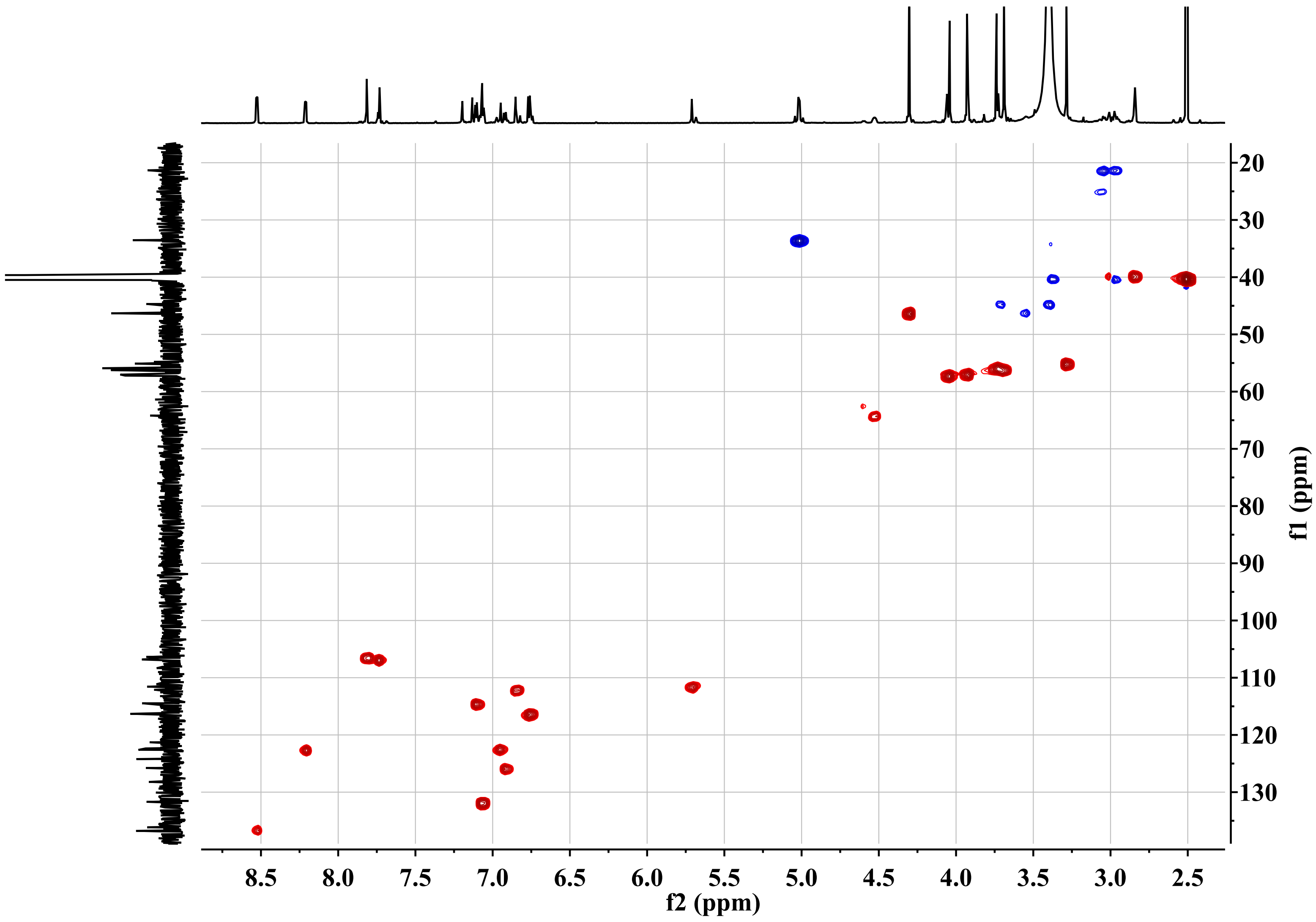


**Figure S10**. HSQC NMR spectrum of (*R*)-nomimantharine trifluoroacetate (**2**)

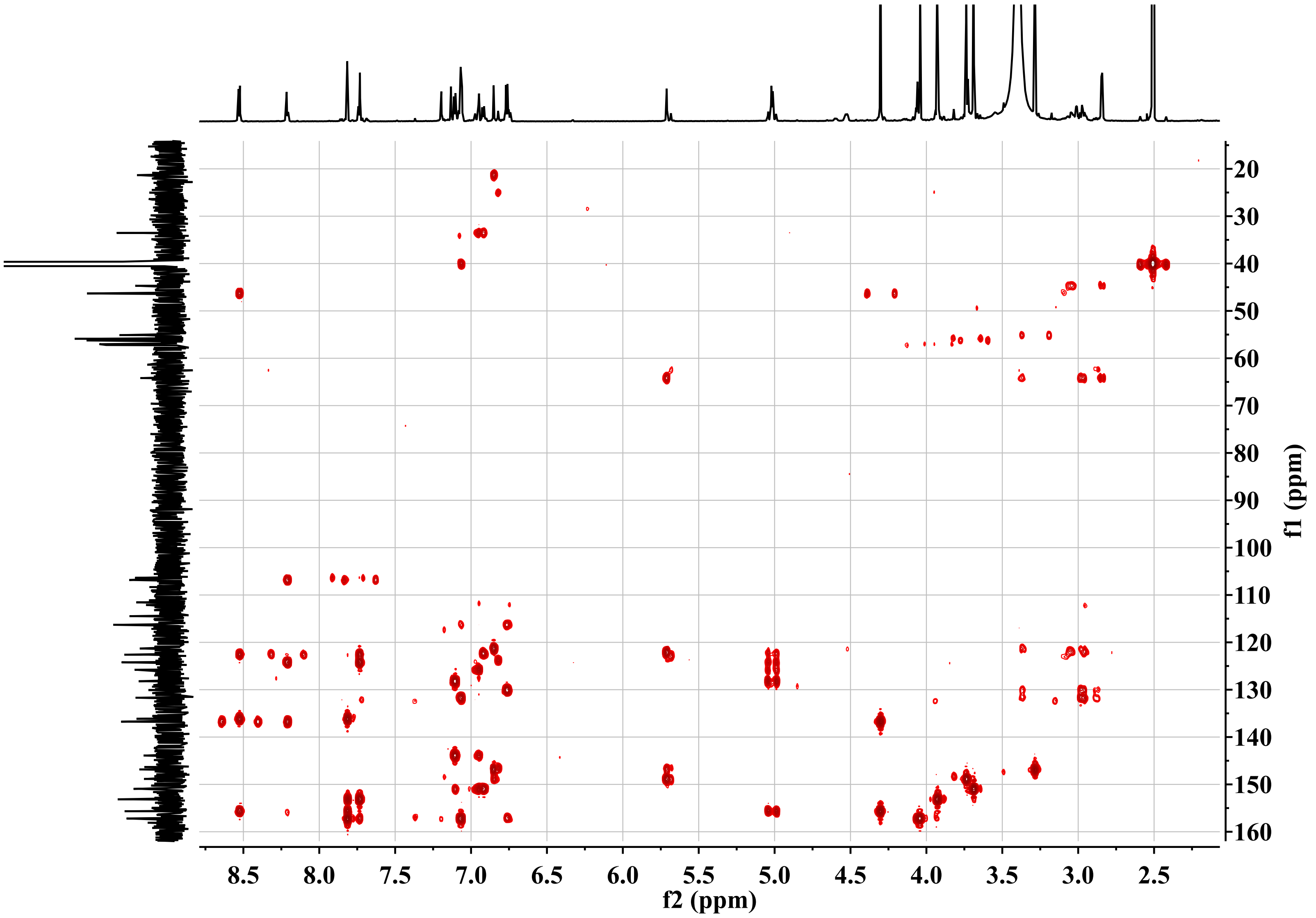


**Figure S11**. HMBC NMR spectrum of (*R*)-nomimantharine trifluoroacetate (**2**)

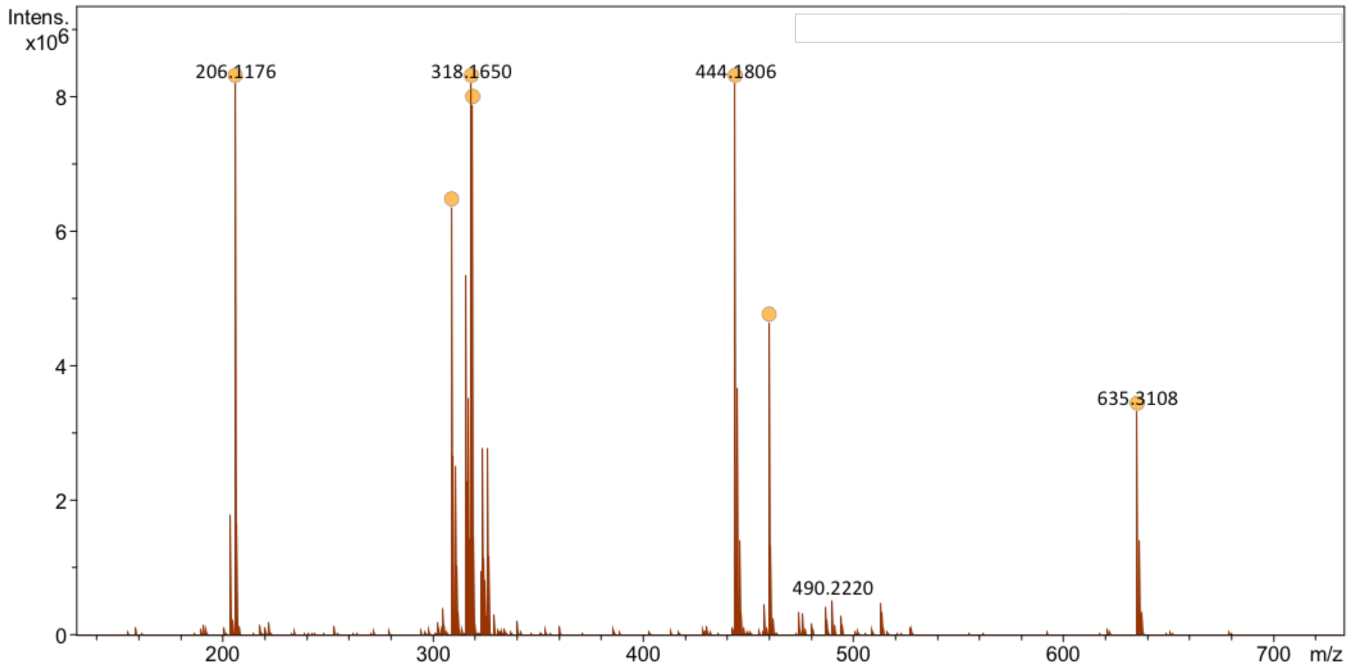


| Means. m/z | Ion formula | calc. m/z | err [ppm] | rdb | e⁻Conf | N-Rule |
|---|---|---|---|---|---|---|
| 635.3108 | $C_{39}H_{43}N_2O_6$ | 635.3116 | 1.2 | 19.5 | even | ok |

**Figure S12**. HRMS spectrum of (*R*)-nomimantharine trifluoroacetate (**2**)

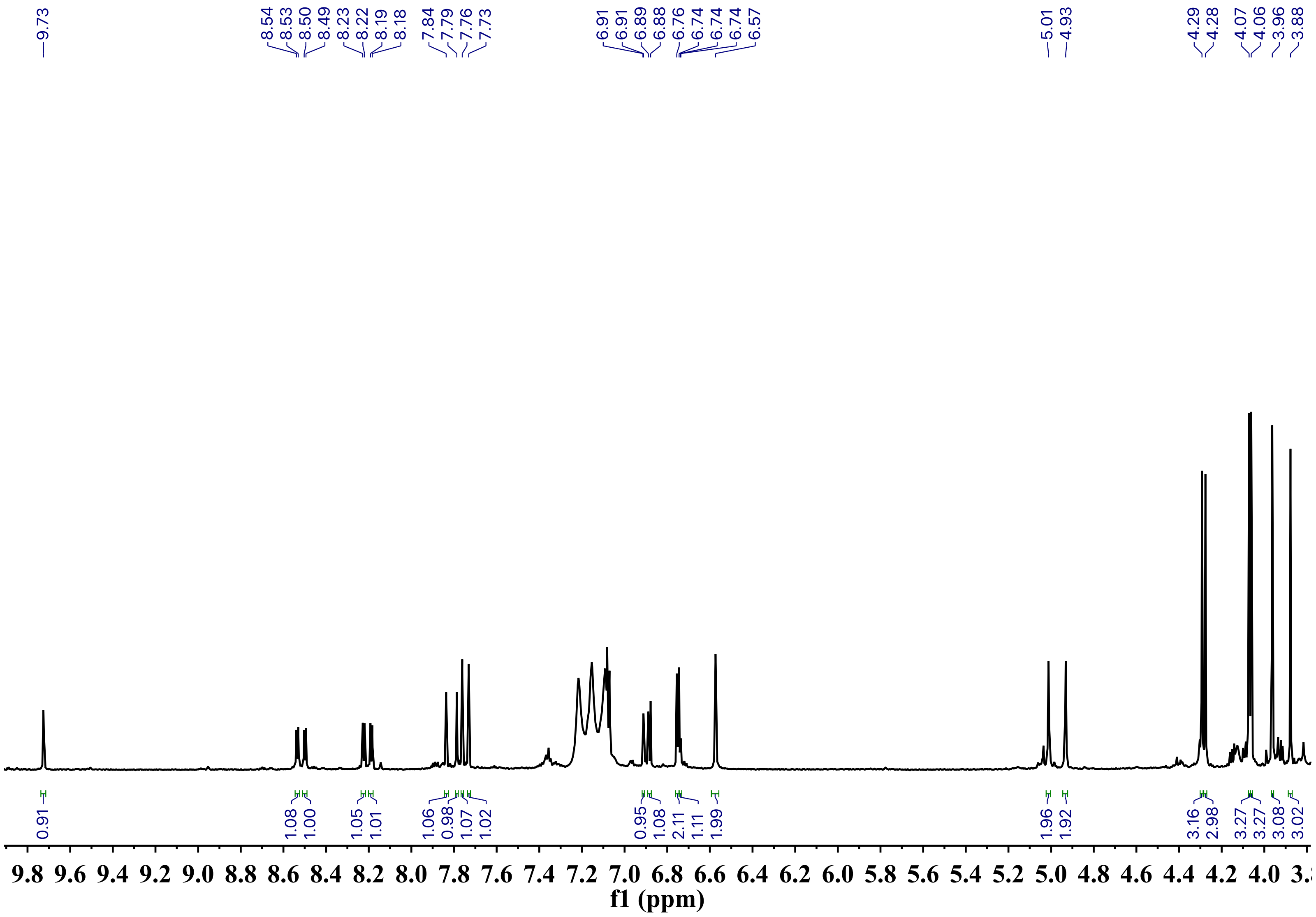


**Figure S13**. $^{1}$H NMR spectrum of 12-de-*O*-methyphaeantharine trifluoroacetate (**3**)

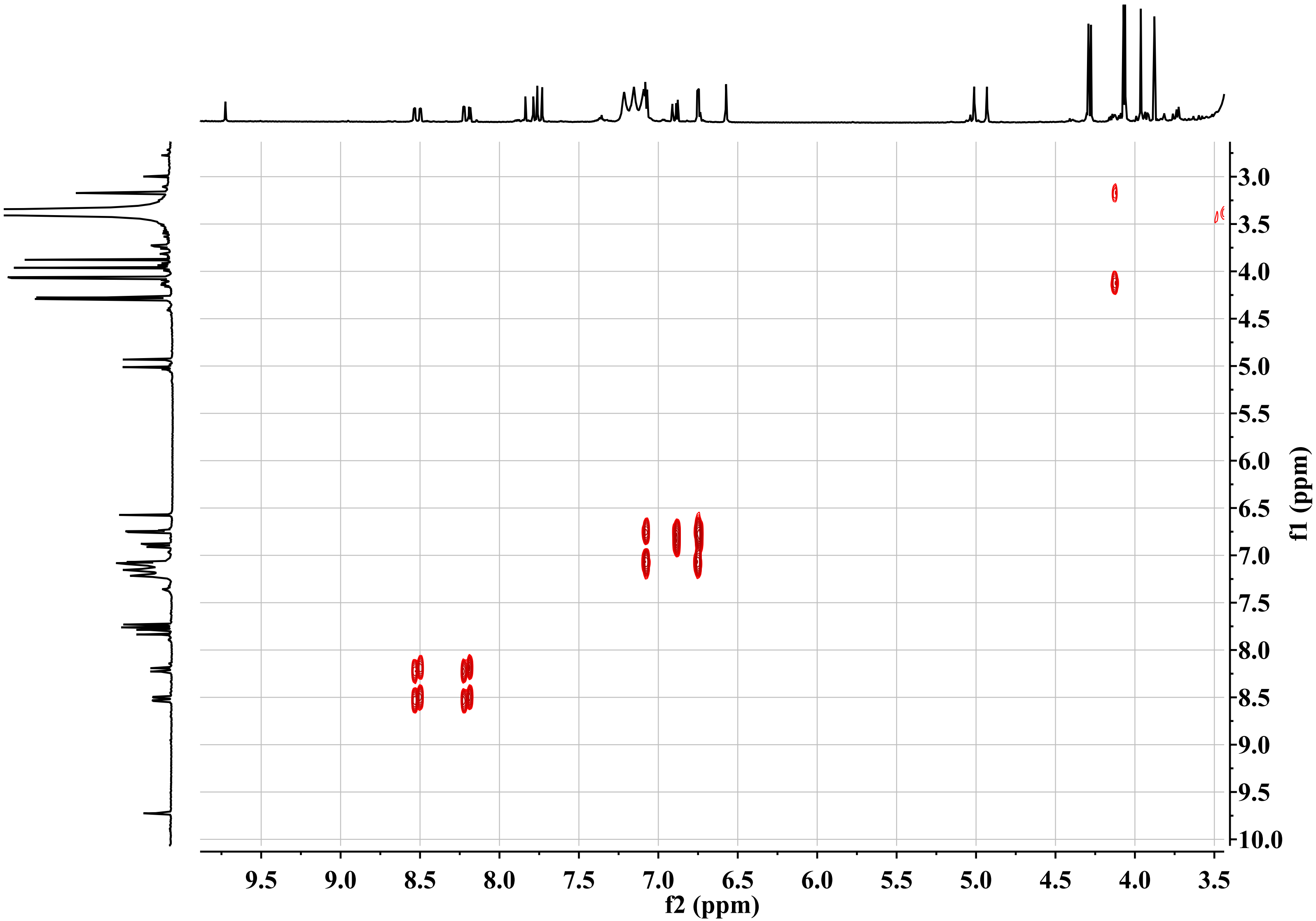


**Figure S14**. COSY NMR spectrum of 12-de-*O*-methyphaeantharine trifluoroacetate (**3**)

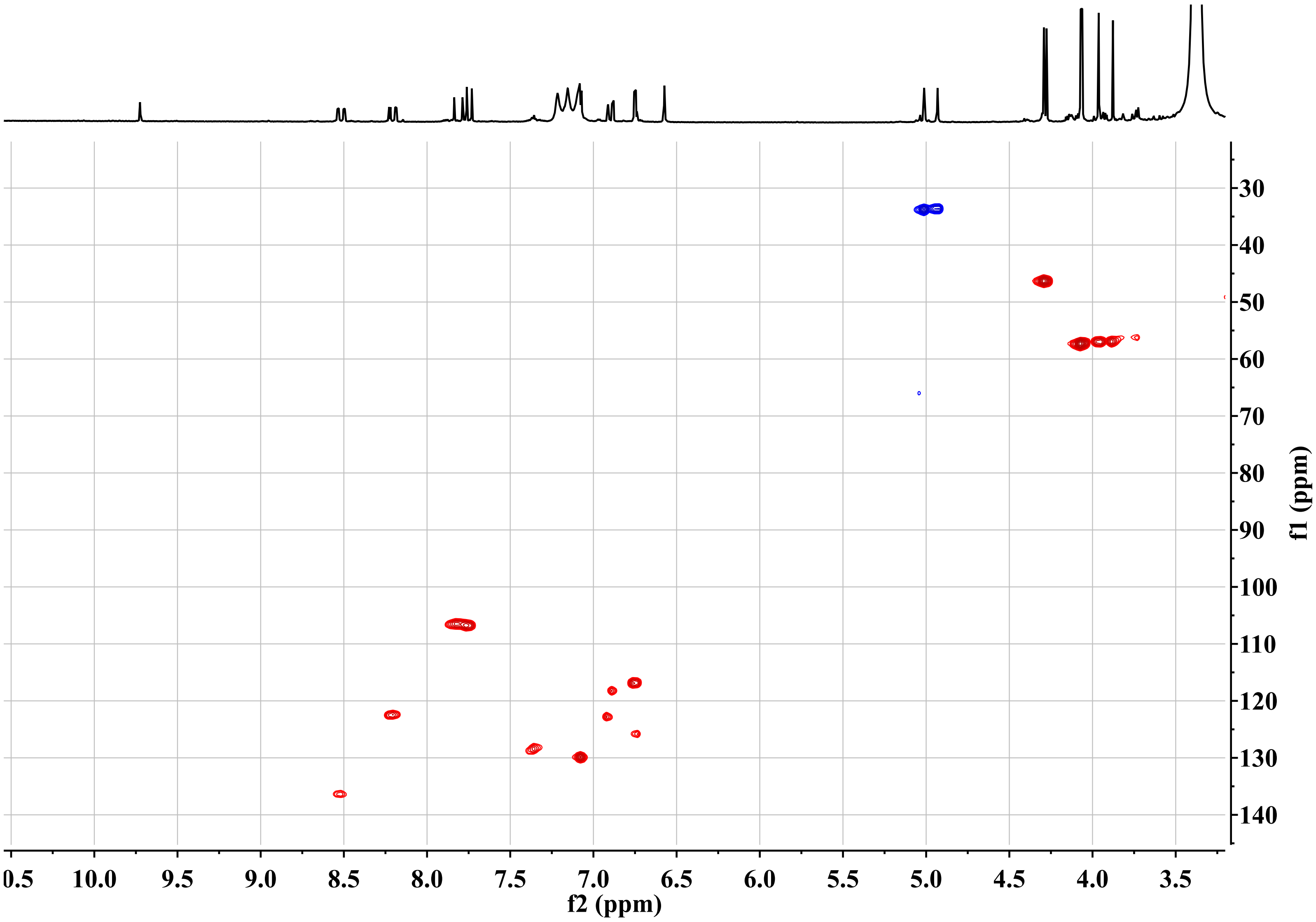


**Figure S15**. HSQC NMR spectrum of 12-de-*O*-methyphaeantharine trifluoroacetate (**3**)

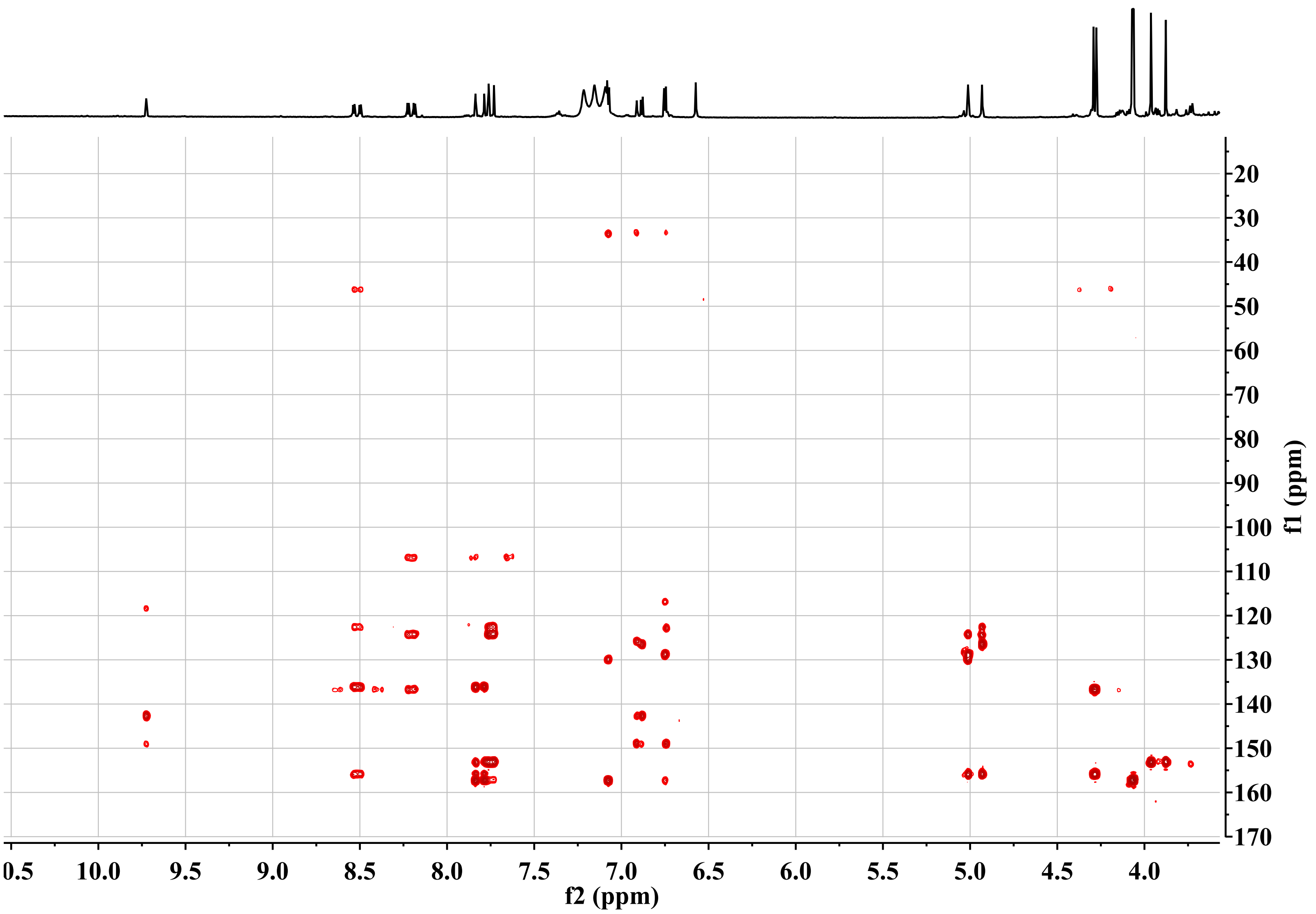


**Figure S16**. HMBC NMR spectrum of 12-de-*O*-methyphaeantharine trifluoroacetate (**3**)

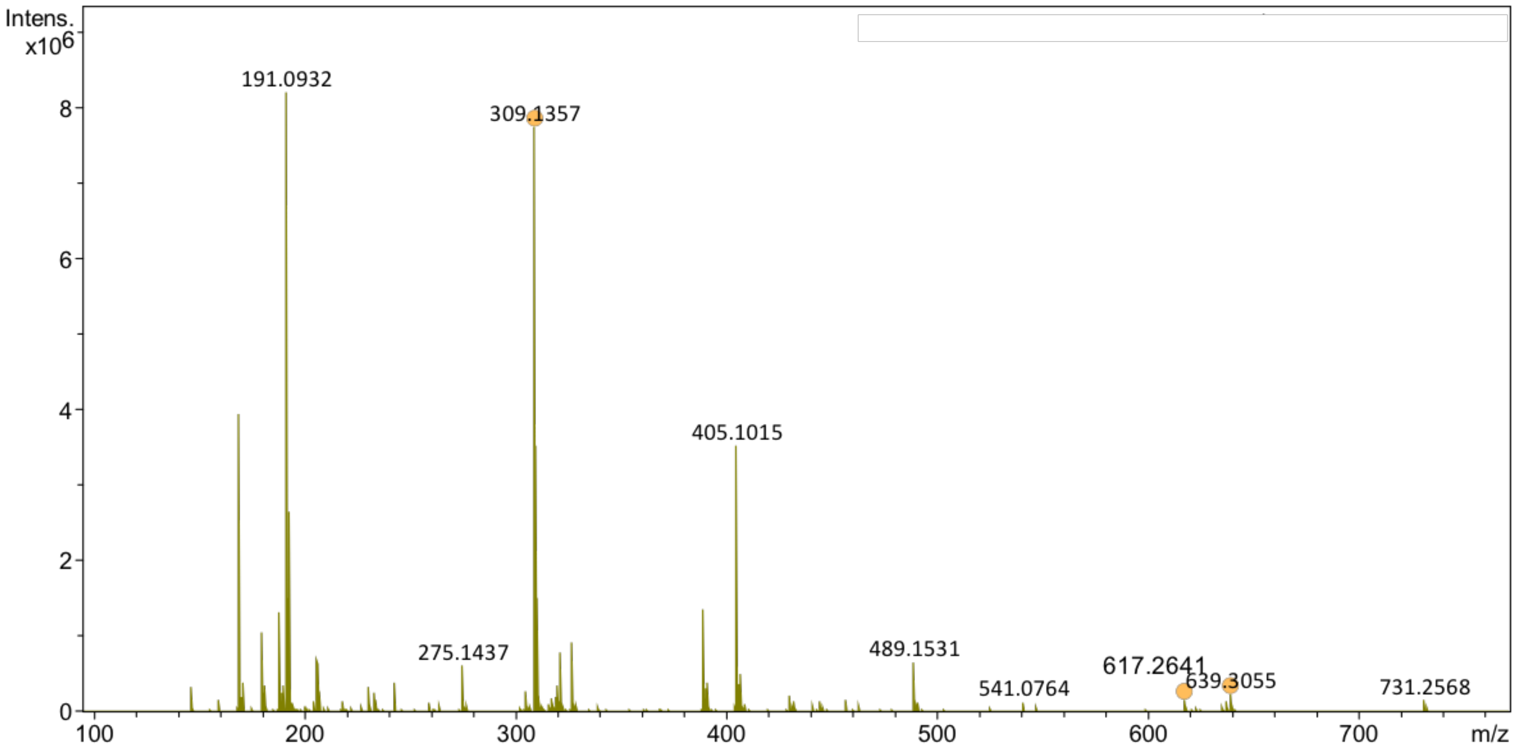


| Means. m/z | Ion formula | calc. m/z | err [ppm] | rdb | e⁻Conf | N-Rule |
|---|---|---|---|---|---|---|
| 309.1357 | $C_{38}H_{38}N_2O_6$ | 309.1359 | 0.6 | 21.0 | even | ok |
| 617.2641 | $C_{38}H_{37}N_2O_6$ | 617.2646 | -1.6 | 21.5 | even | ok |

**Figure S17**. HRMS spectrum of 12-de-*O*-methyphaeantharine trifluoroacetate (**3**)

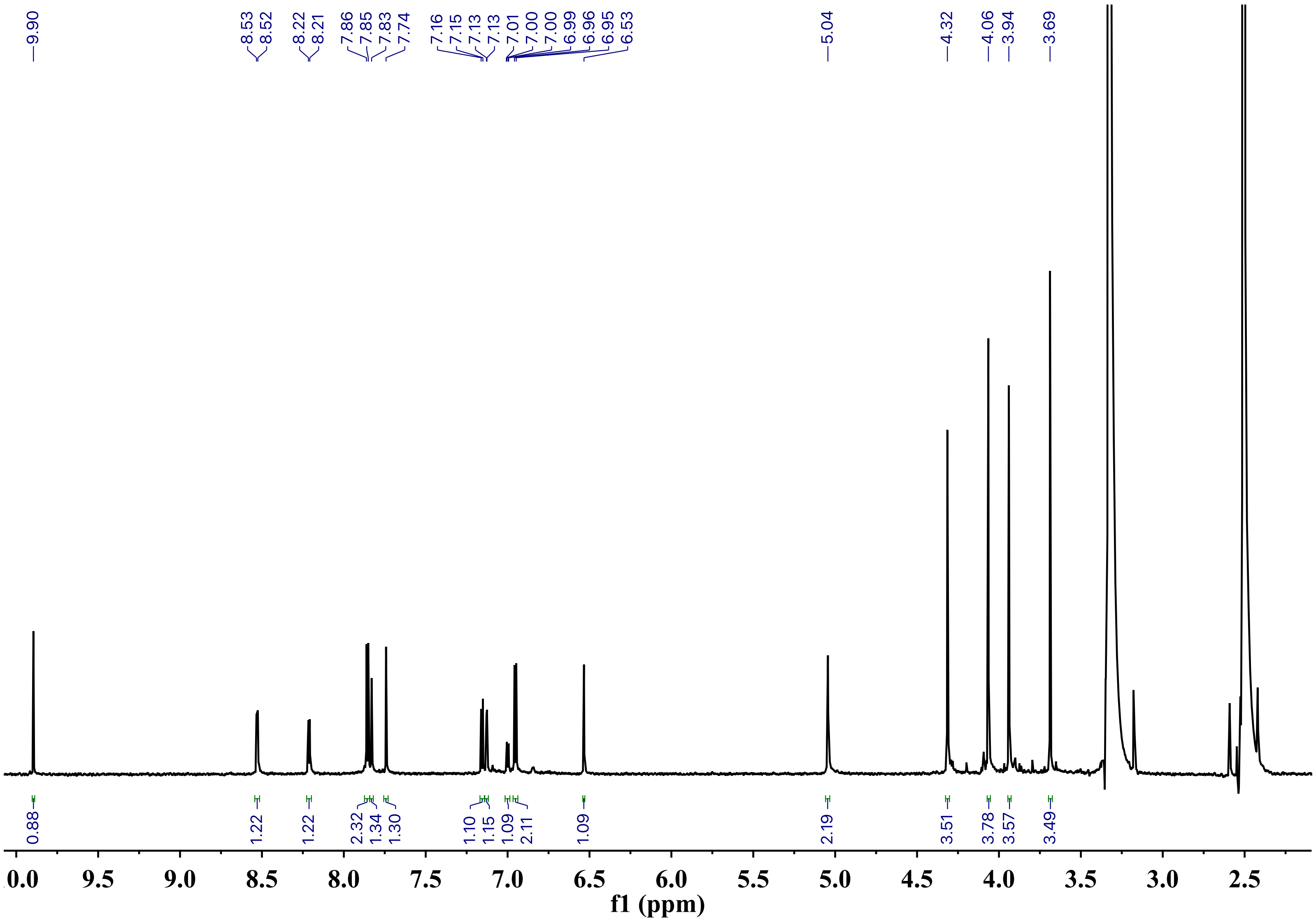


**Figure S18**. $^{1}$H NMR spectrum of nominanthranal trifluoroacetate (**4**)

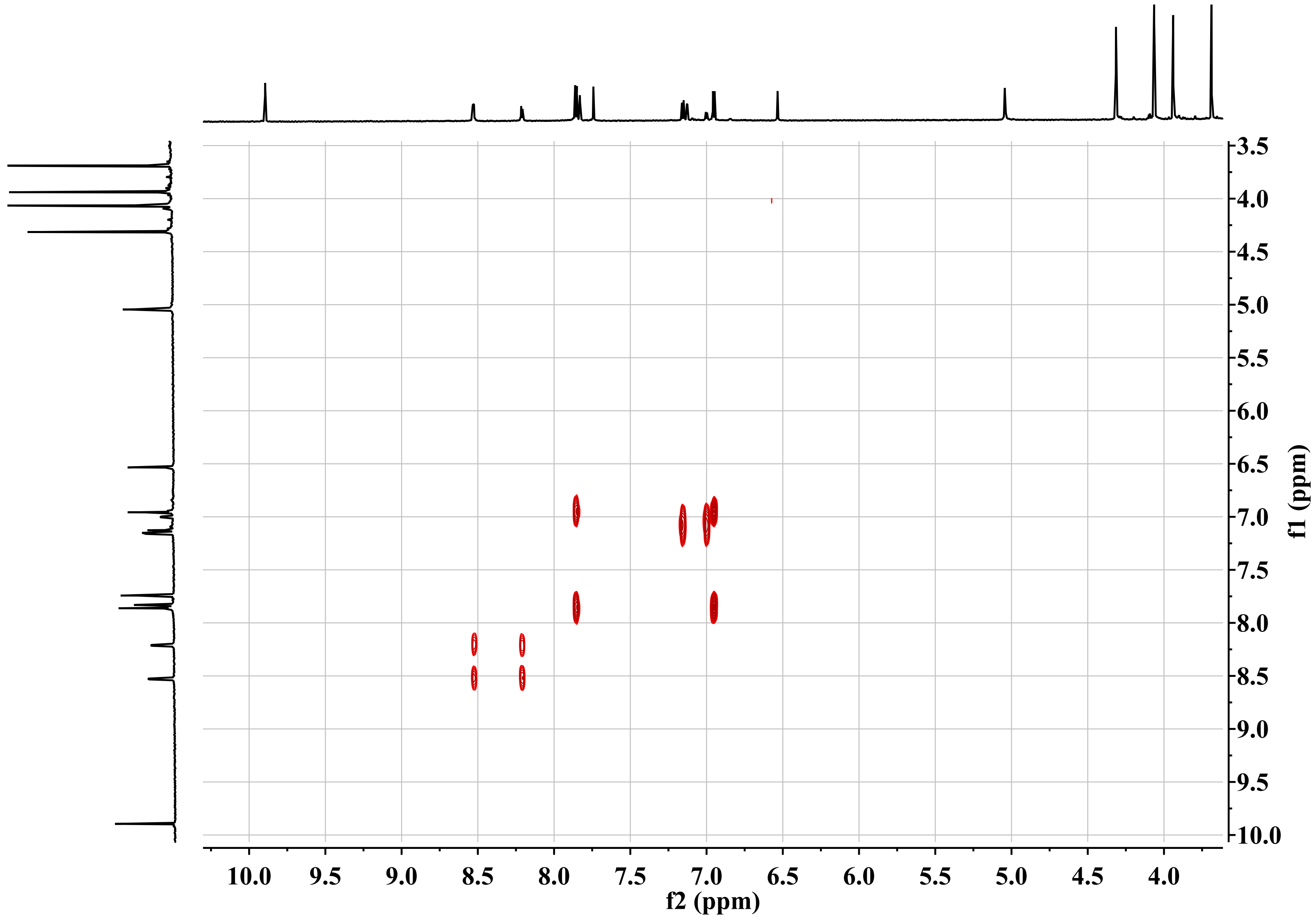


**Figure S19**. COSY NMR spectrum of nominanthranal trifluoroacetate (**4**)

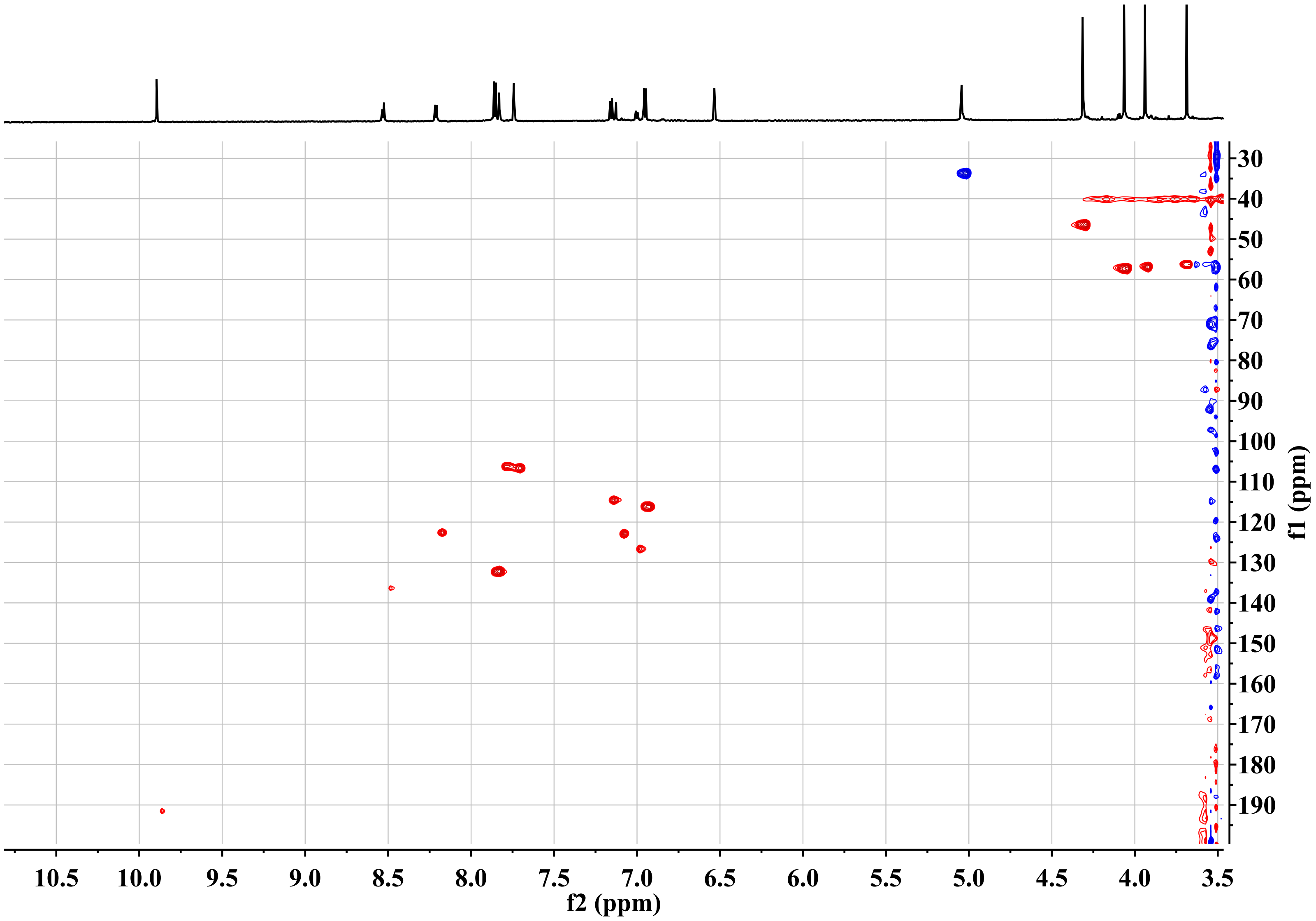


**Figure S19**. HSQC NMR spectrum of nominanthranal trifluoroacetate (**4**)

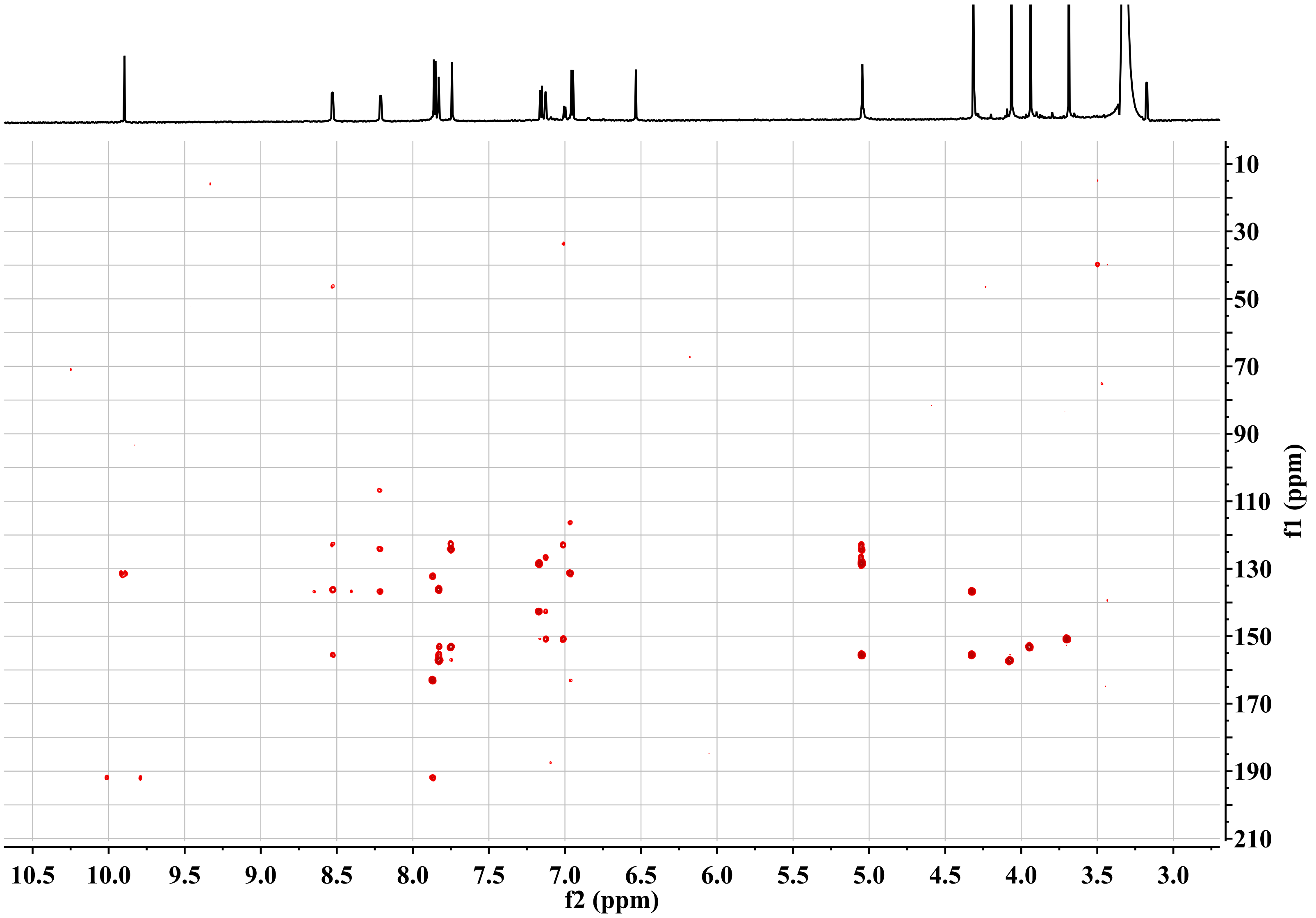


**Figure S20**. HMBC NMR spectrum of nominanthranal trifluoroacetate (**4**)

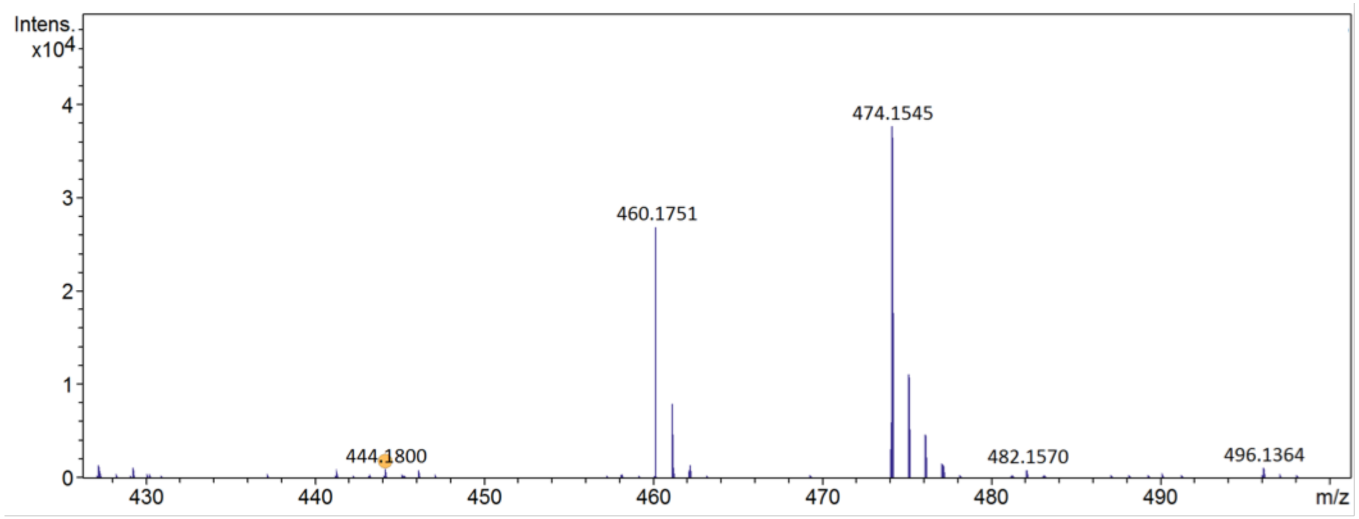


| Means. m/z | Ion formula | calc. m/z | err [ppm] | rdb | e⁻Conf | N-Rule |
|---|---|---|---|---|---|---|
| 444.1800 | $C_{27}H_{26}NO_5$ | 444.1805 | 1.2 | 15.5 | even | ok |

**Figure S21**. HRMS spectrum of nominanthranal trifluoroacetate (**4**)

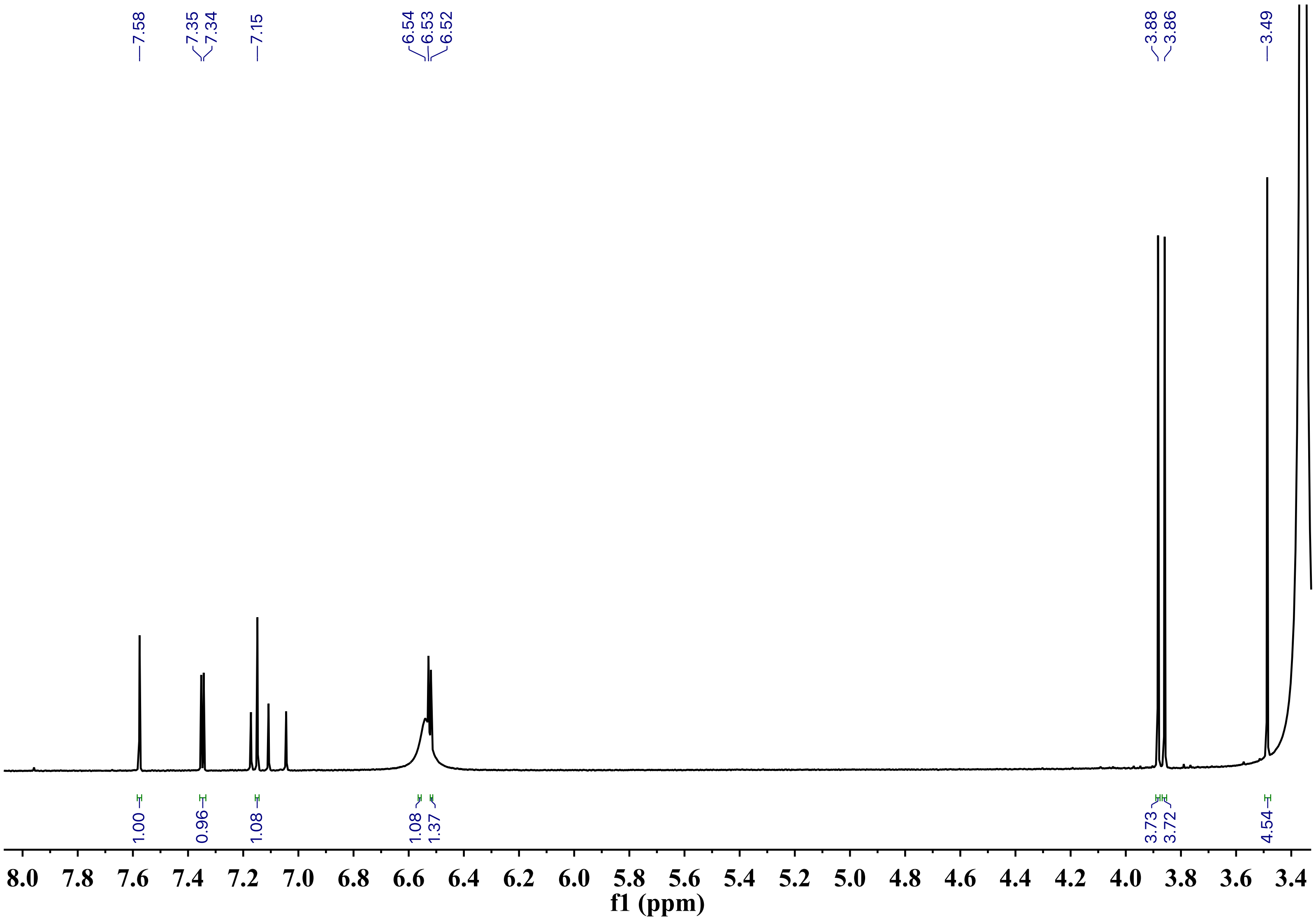


**Figure S22**. $^{1}H$ NMR spectrum of 1-hydroxy-6,7-dimethoxy-2-methylisoquinoline trifluoroacetate (**5**)

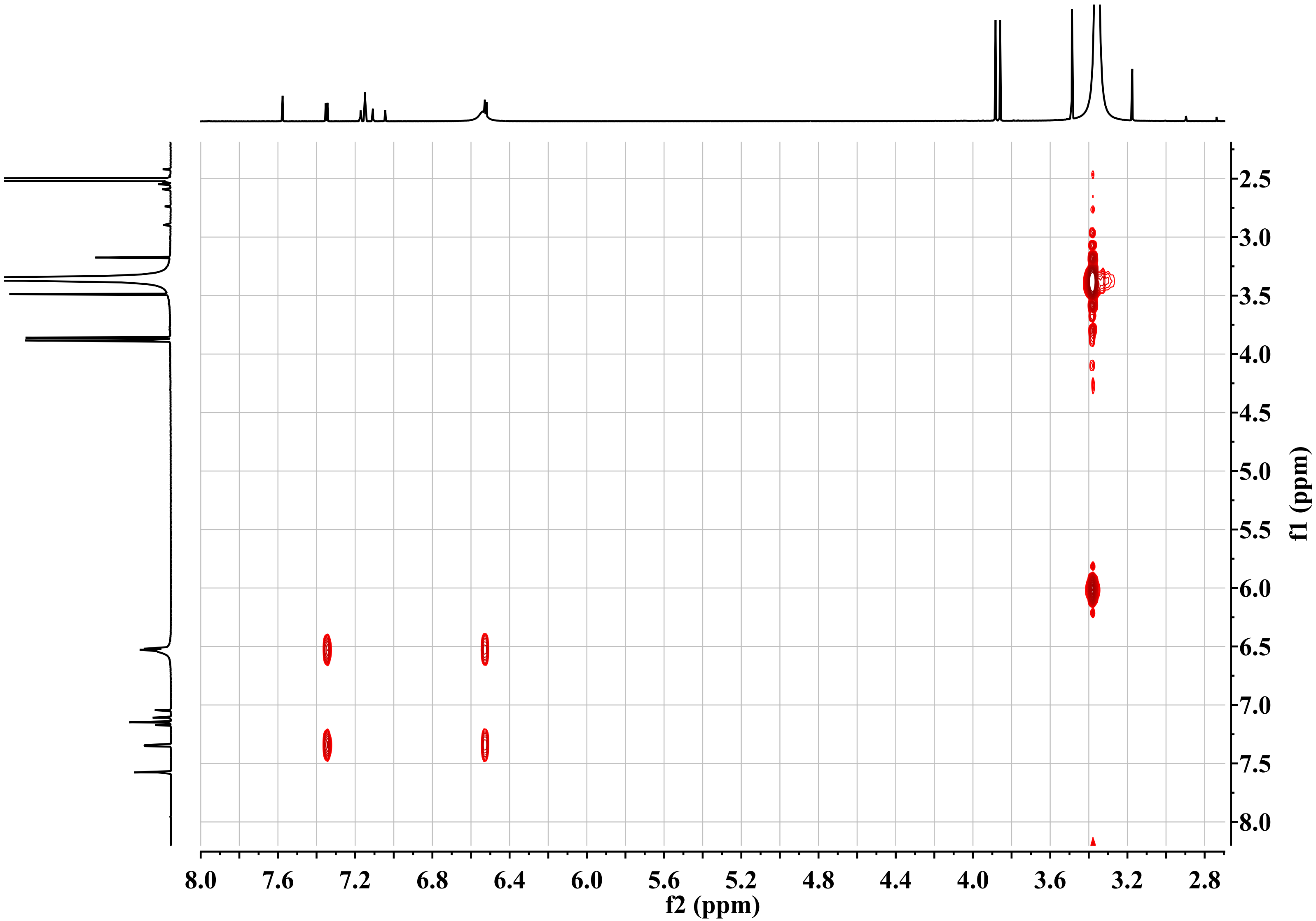


**Figure S23**. COSY NMR spectrum of 1-hydroxy-6,7-dimethoxy-2-methylisoquinoline trifluoroacetate (**5**)

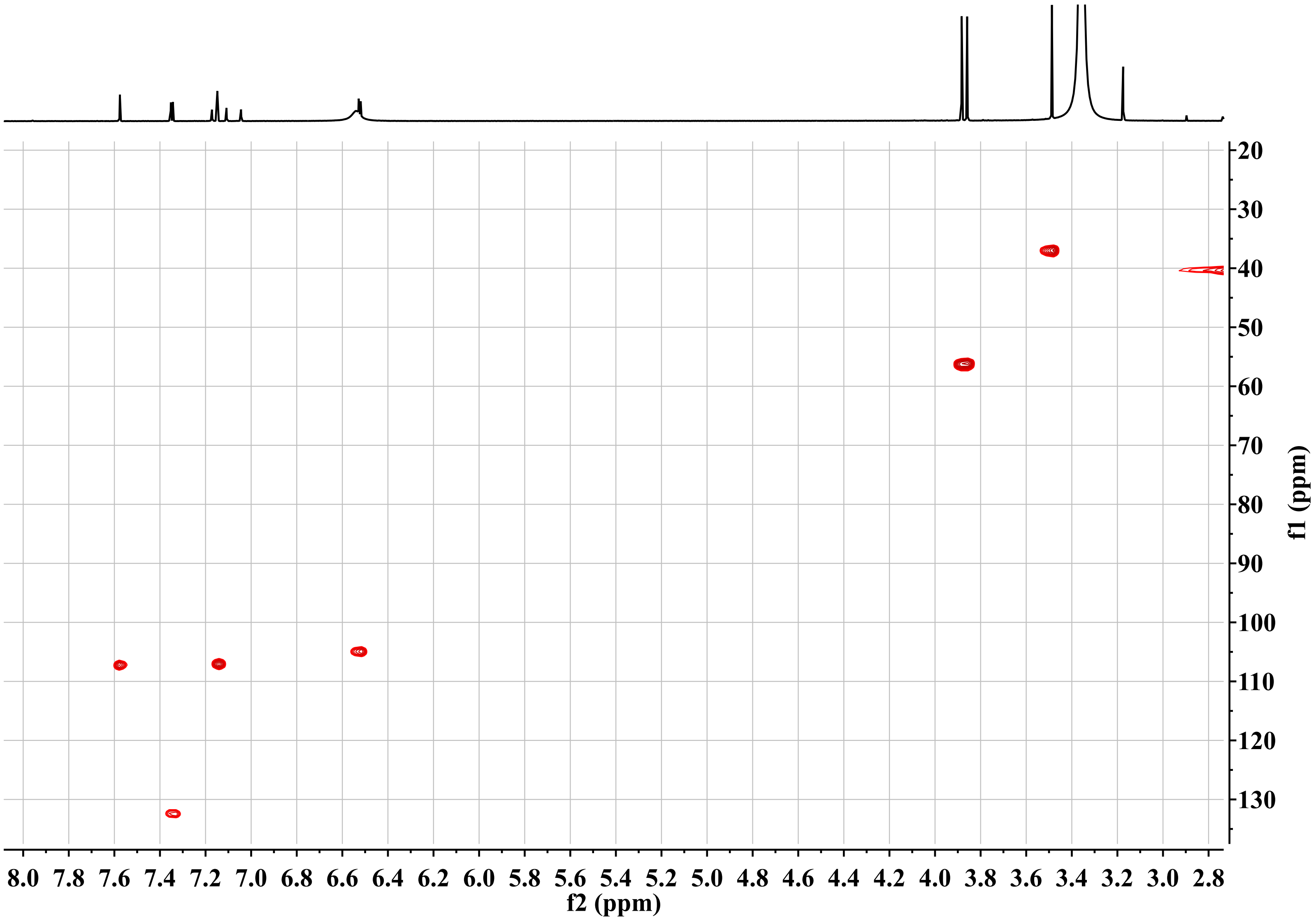


**Figure S24**. HSQC NMR spectrum of 1-hydroxy-6,7-dimethoxy-2-methylisoquinoline trifluoroacetate (**5**)

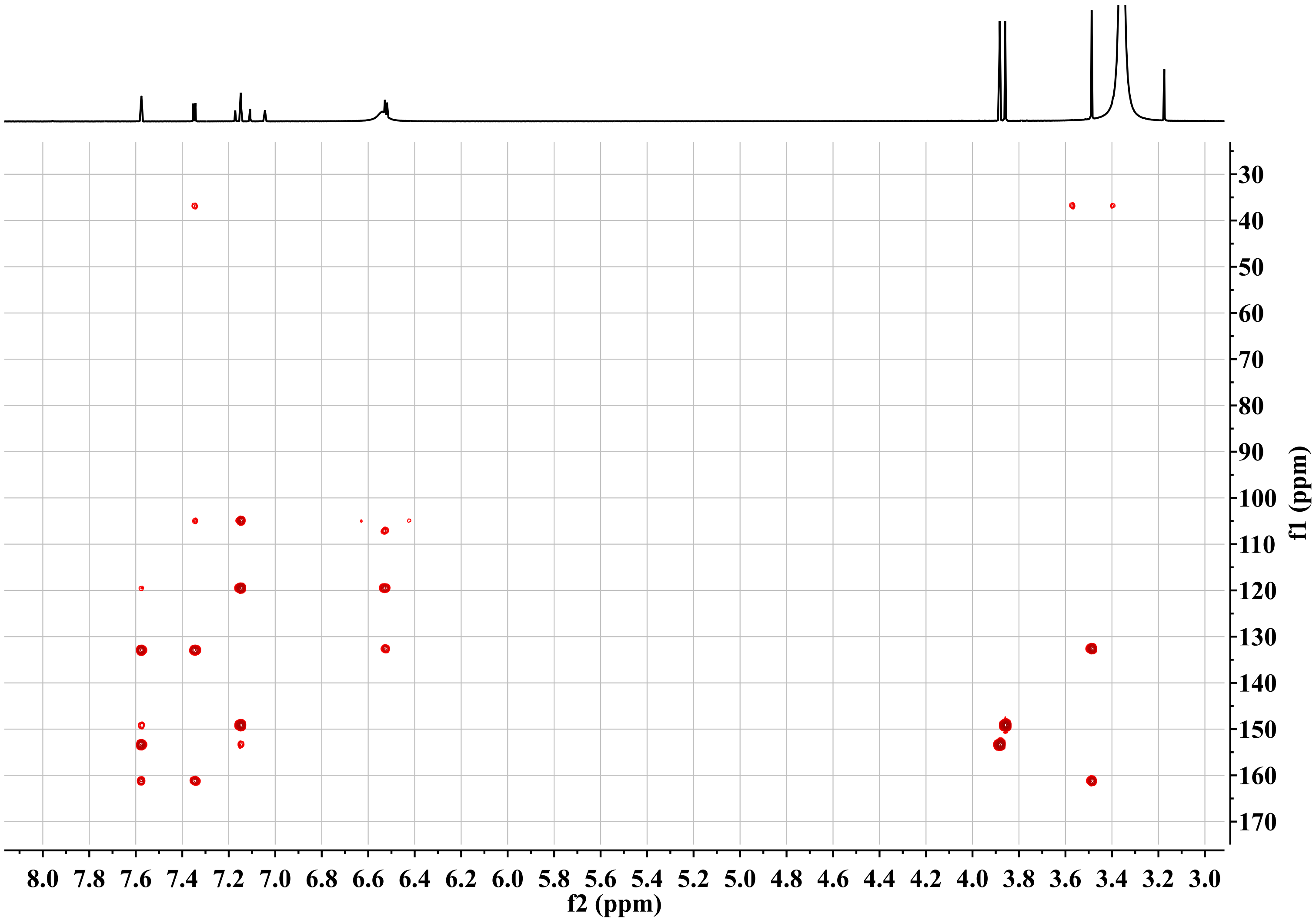


**Figure S25**. HMBC NMR spectrum of 1-hydroxy-6,7-dimethoxy-2-methylisoquinoline trifluoroacetate (**5**)

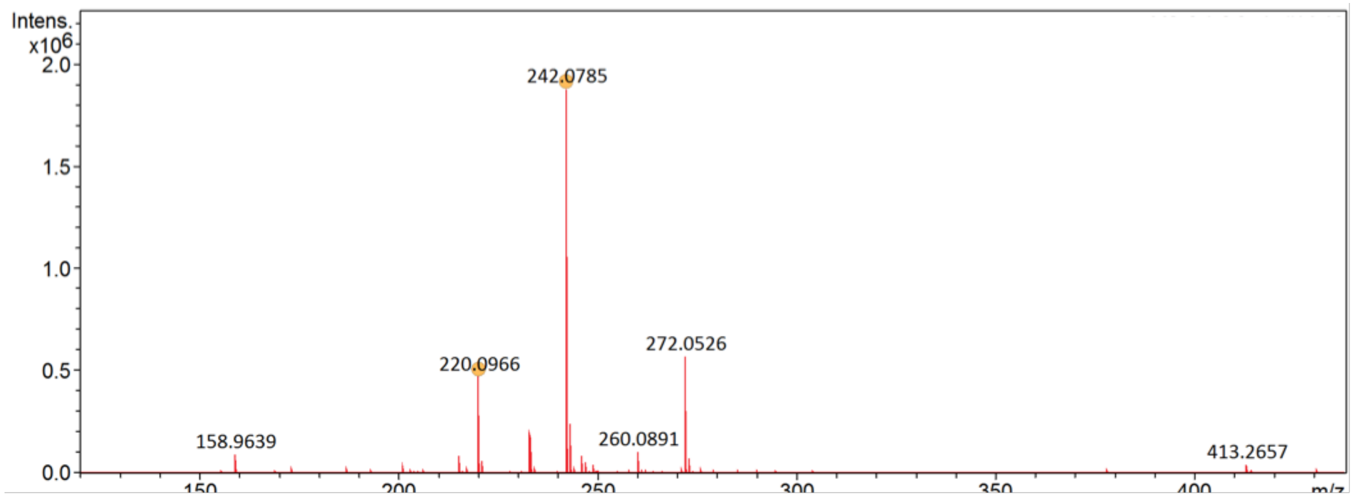


| Means. m/z | Ion formula | calc. m/z | err [ppm] | rdb | e⁻Conf | N-Rule |
|---|---|---|---|---|---|---|
| 220.0966 | $C_{12}H_{14}NO_3$ | 220.0968 | 1.0 | 6.5 | even | ok |
| 242.0785 | $C_{12}H_{13}NNaO_3$ | 242.0788 | 1.1 | 6.5 | even | ok |

**Figure S26**. HRMS spectrum of 1-hydroxy-6,7-dimethoxy-2-methylisoquinoline trifluoroacetate (**5**)

Table S1. Stable conformers of (*R*)-nomimantharine trifluoroacetate (**2**)

| Conformer | Conformation | Energy (kcal/mol) | Percent (%) |
| --- | --- | --- | --- |
| **2**-1 | 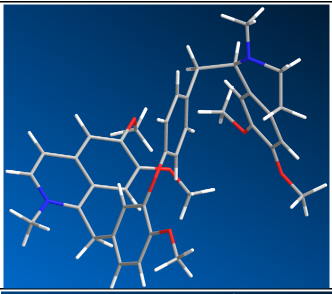 | -1300666.800 | 67.3 |
| **2**-2 | 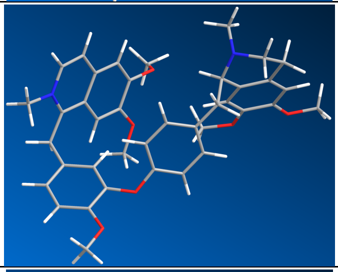 | -1300665.662 | 9.8 |
| **2**-3 | 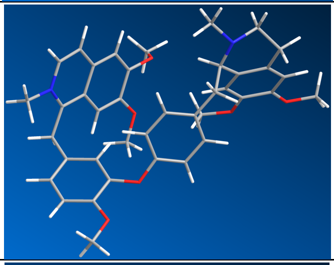 | -1300665.576 | 8.5 |
| **2**-4 | 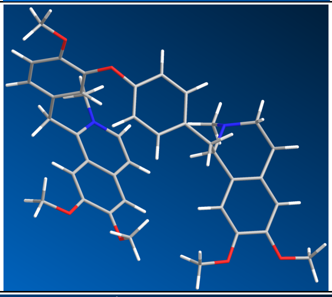 | -1300665.506 | 7.5 |
| **2**-5 | 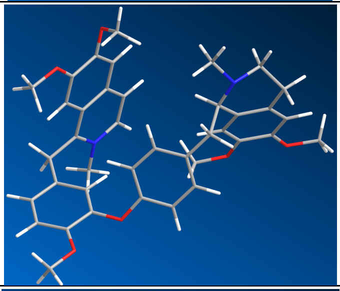 | -1300665.079 | 3.7 |
| **2**-6 | 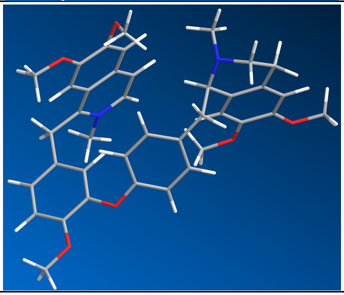 | -1300664.989 | 3.2 |
